# Mg(II) Coordination Polymers Based on Flexible Isomeric Tetracarboxylate Ligands: Syntheses, Structures, Structural Transformation and Luminescent Properties

**DOI:** 10.3390/polym10040371

**Published:** 2018-03-26

**Authors:** Kedar Bahadur Thapa, Xiang-Kai Yang, Jhy-Der Chen

**Affiliations:** Department of Chemistry, Chung-Yuan Christian University, Chung-Li 320, Taiwan; kedar.bthapa1977@gmail.com (K.B.T.); xiangkaishulin@gmail.com (X.-K.Y.)

**Keywords:** coordination polymer, tetracarboxylate, X-ray structure, structural transformation

## Abstract

By using a new flexible tetracarboxylic acid, bis(3,5-dicarboxyphenyl) adipoamide, H_4_**L**^1^, and its isomer, bis(2,5-dicarboxyphenyl)adipoamide, H_4_**L**^2^, three Mg(II) coordination polymers, {[Mg_2_(**L**^1^)(H_2_O)_2_]·2EtOH·3H_2_O}_n_, **1**, [Mg_2_(**L**^1^)(H_2_O)_8_]_n_, **2**, and {[Mg_2_(**L**^2^)(H_2_O)_6_]·H_2_O}_n_, **3**, have been hydrothermally synthesized and structurally characterized by single-crystal X-ray diffraction. Complexes **1** and **2** are the solvent ratio-dependent hydrothermally stable products. The tetracarboxylate ligand of complex **1** connects eight Mg(II) ions through nine oxygen atoms, resulting in a three-dimensional (3D) 5-connected uninodal net with a rare non-interpenetrating (4^4^.6^6^)-pcu-5-Pmna topology, whereas those of **2** and **3** link four Mg(II) ions through four oxygen atoms and six Mg(II) ions through six oxygen atoms, forming a 1D linear chain and a 3,6-connected 2-nodal 3D net having {4.6^2^}_2_{4^2^.6^10^.8^3^}-**rtl** topology, respectively. Complex **1** shows a series of structural transformations on heating to 200 °C and almost reversible structural transformation when the activated products were immersed in a mixture of ethanol and water or on hydrothermal. Likewise, complex **2** exhibits a reversible structural transformation on heating/hydrothermal, while **3** exhibits irreversible structural transformations. All three complexes exhibit blue light emissions, with that of complex **3** being much more intense.

## 1. Introduction

The investigation of coordination polymers (CPs) continues to be an intense area of research activity due to their intriguing structural topologies and promising potential applications as multifunctional materials [1,2,3,4,5,6,7,8]. The structural types of the resulting CPs are influenced by various factors, such as organic ligands, metal ions, counterions, metal-to-ligand ratio, solvent system, and the temperature and pH values of the solution, even though the control of the desired structural dimensionality of CPs remains a challenge [7,8]. Generally, the structural transformations of CPs can be induced by various methods, including external stimuli like heat, light and mechanical forces, removal and uptake of solvents, exposure to reactive vapors, and exchange of solvents and guest molecules, which are rare due to the breaking and forming of coordinate and/or covalent bonds in multiple directions [9,10]. The applications of such phenomena observed in CPs are significant for sensors and switches.

The construction of CPs from s-block metal centers has been explored to a lesser extent than in transition metals (TMs), and their coordination ability is inferior compared with that of TMs; furthermore, they are lighter (lower atomic weight) and bigger in size, and are filled shell orbitals in ionic form, which makes them difficult incorporate into CPs [11,12,13]. Among the alkali-earth metals, the Mg^2+^ ions deserve more attention and have great flexibility in the construction of CPs because they are not only recognized as abundant, non-toxic and low-cost metal ions, but also have high polarizing power for strong coordination bonds with oxygen; thus, Mg-O bonds consist of a good mixture of covalence and ionicity.

**Scheme 1:**
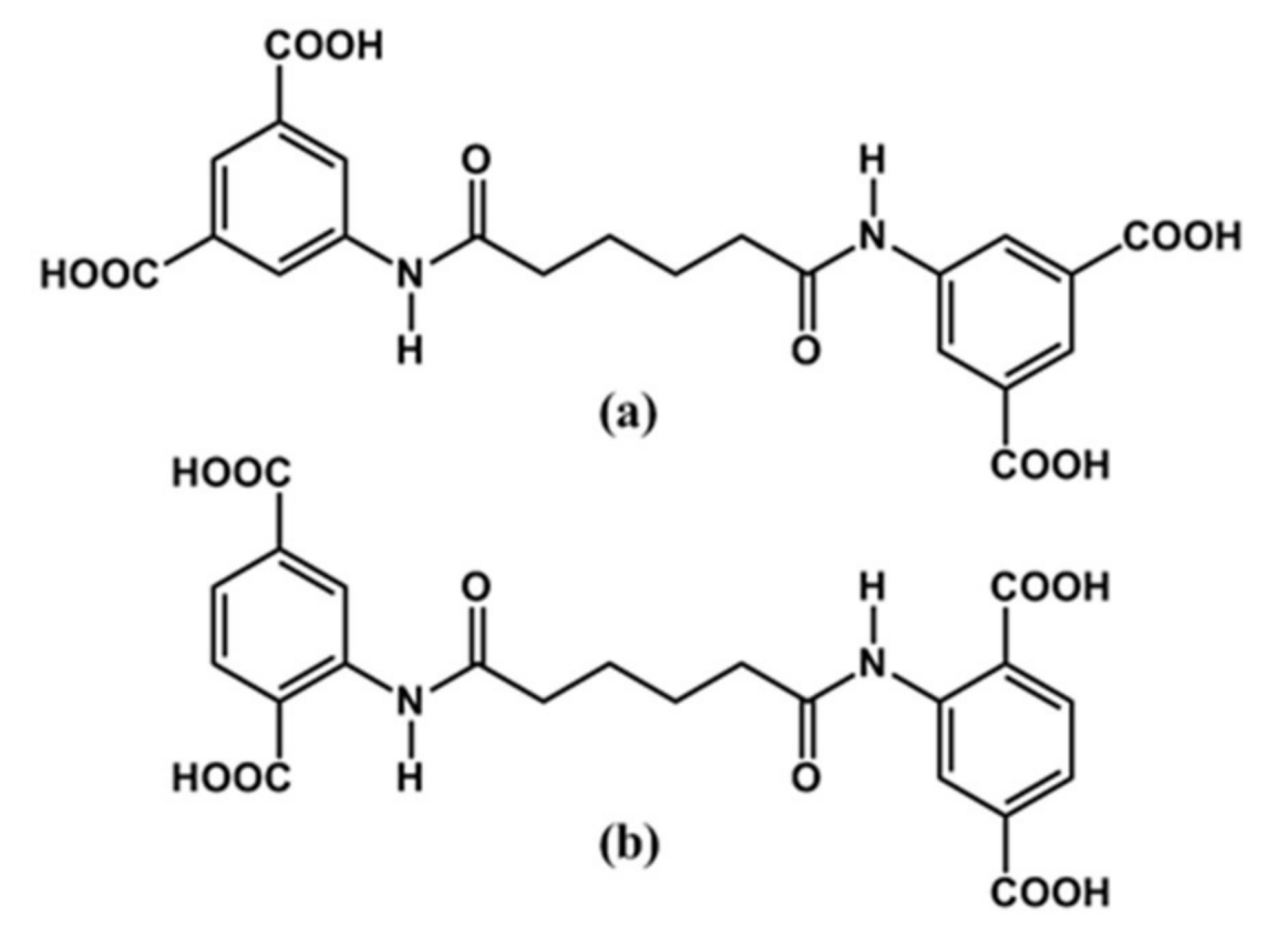
Schematic drawings for (**a**) H_4_**L**^1^ and (**b)** H_4_**L**^2^

Tetracarboxylate ligands have been widely used as linkers for the assembly of various CPs [14,15,16,17,18,19,20,21,22,23,24], but the involvement of these ligands in the construction of Mg(II) CPs has very rarely been explored [25,26]. Only rigid and semi-rigid di-, tri- or tetra-carboxylate ligands are commonly used for the synthesis of Mg(II) CPs; however, the flexible tetracarboxylate ligand, to the best of our knowledge, has not yet been explored for the construction of Mg(II) CPs [27,28,29,30,31]. In addition, tetracarboxylate ligands have strong coordination abilities in various modes with alkali-earth metals that satisfy the requirements of the coordination geometries of metal centers. As a result, they are capable of building diverse multidimensional architectures. Based on the above concerns, we have synthesized a new bis(3,5-dicarboxyphenyl)adipoamide ligand, H_4_**L**^1^, Scheme 1a, which has four carboxylate groups as coordination sites, as well as its isomeric compound bis(2,5-dicarboxyphenyl)adipoamide, H_4_**L**^2^, Scheme 1b, as it is important to study the ligand-based isomeric effect on the structures and properties of Mg(II) CPs.

**Scheme 2:**
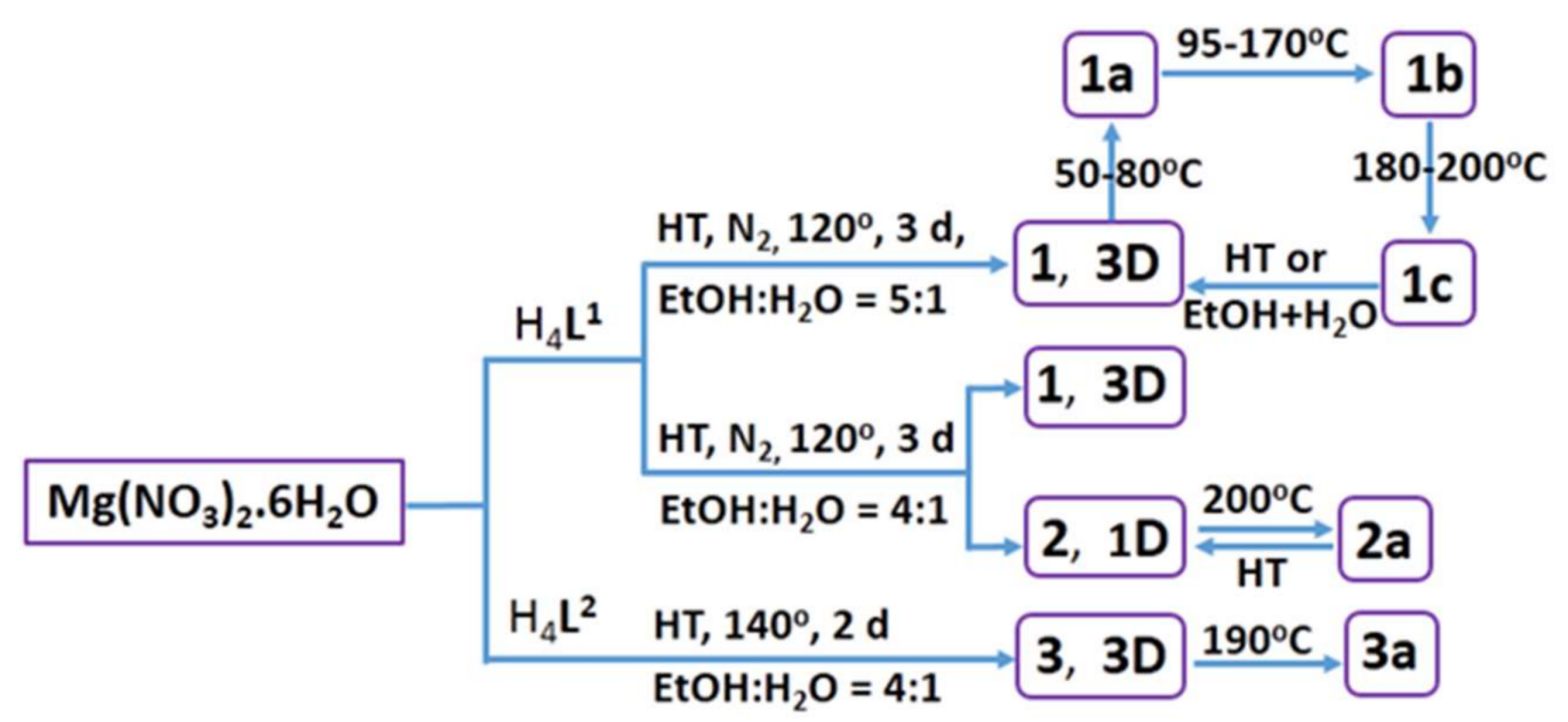
Overall sketch for complexes **1**–**3**.

Herein, we report three Mg(II) CPs, {[Mg_2_(**L**^1^)(H_2_O)_2_]·2EtOH·3H_2_O}_n_, **1**; [Mg_2_(**L**^1^)(H_2_O)_8_]_n_, **2**; and {[Mg_2_(**L**^2^)(H_2_O)_6_]·H_2_O}_n_, **3**, having the different dimensional architectures, in which complexes **1** and **2** are the solvent ratio-dependent products. Additionally, the tetracarboxylate ligands of **1**–**3** connect eight, four and six Mg(II) ions, respectively. Moreover, all these complexes display structural transformations on heating, and Complex **3** shows a much more intense blue photoluminescence than the other two complexes. Scheme 2 presents the overall sketch for complexes **1**–**3**.

## 2. Materials and Methods

### 2.1. General Procedures

Solvents were dried and deoxygenated by refluxing over the appropriate reagents before use. Elemental analyses were obtained from a PE 2400 series II CHNS/O analyzer (PerkinElmer, Boston, MA, USA) or an Elementar Vario EL cube analyzer (Elementar Americas Inc., Ronkonkoma, NJ, USA). ^1^H-NMR and TOF-MS (ESI-MS) were recorded on a Bruker 400 MHz NMR and a Bruker MicrOTOF II (Bruker, Billerica, MA, USA), respectively. Powder X-ray diffraction patterns were obtained from a Bruker D2 PHASER diffractometer (Bruker, Billerica, MA, USA) with CuK_α_ (λ_α_ = 1.54 Å) radiation. IR spectra (KBr disk) were recorded on a Jasco FT/IR-460 plus spectrometer (JASCO, 28600 Mary’s Court City, MD, USA). The UV-Vis absorption spectra and emission spectra were obtained in the solid state at room temperature by using an SP-1901 UV-Vis spectrophotometer (Shimadzu, Kyoto, Japan) and a Hitachi F-4500 spectrometer (Hitachi, Tokyo, Japan), respectively. Thermal gravimetric analysis (TGA) was carried out on a SII EXSTAR6000 TG/DTA 6200 (Seiko Instruments Inc., Tokyo, Japan) under N_2_ atmosphere at a heating rate of 10 °C min^−1^ in the temperature range of 30 to 1000 °C. 

### 2.2. Materials

The reagent Mg(NO_3_)_2_·6H_2_O was purchased from Alfa Aesar Co., (Heysham, UK). The other reagents 5-aminoisophthalic acid, 2-aminoterephthalic acid and adipoyl chloride were obtained from ACROS Organics (Geel, Belgium). The bis-(2,5-dicarboxyphenyl)adipoamide, H_4_**L**^2^ was synthesized in one step using a modified version of a previously reported procedure [14].

### 2.3. Preparations

#### 2.3.1. Bis(3,5-dicarboxyphenyl)adipoamide, H_4_**L**^1^

Adipoyl chloride (1.00 g, 5.47 mmol) in 10 mL DMF was added slowly to a 10 mL DMF solution of 5-amino-isophthalic acid (1.98 g, 10.94 mmol) kept in the reflux flask facilitated with the magnetic stirrer and the mixture was stirred for 30 min. Then, triethylamine (1.11 g, 10.94 mmol) in 10 mL DMF was carefully added dropwise to the stirring solution mixture which was refluxed for one day. After cooling, the mixture was poured in 300 mL water to induce the precipitation. Finally, the solution was filtered, and the residue was washed with 300 mL distilled water to obtain a white powder. Yield: 1.40 g (54%). Anal Calcd for C_22_H_20_N_2_O_10_ (MW = 472.11): C, 55.93; H, 4.27; N, 5.93. Found: C, 55.34; H, 4.25; N, 6.07. ^1^H-NMR (DMSO-*d*_6_, 400 MHz, 25 °C): δ 1.64 (t, 4H; CH_2_), 2.36 (t, 4H; CH_2_), 8.12 (s, 1H; ArH), 8.42 (s, 2H; ArH), 10.26 (s, 2H; NH), Figure S1. ^13^C-NMR (DMSO-*d*_6_, 400 MHz, 25 °C): δ 172.12 (–CONH–), 166.97 (–COOH), 140.31 (NH–isophthalate–C5), 132.12 (NH–isophthalate–C1,3), 124.83 (NH–isophthalate–C2), 123.89 (NH–isophthalate–C4,6), 36.69 (–CH_2_– bonded to amide), 25.10 (–CH_2_– aliphatic), Figure S2. TOF, ESI-MS methalnol (-): *m*/*z* 471.11 [M − H]^−^, Figure S3. IR (cm^−1^): 3734.48 (w), 3355.53 (m), 3114.47 (w), 2960.2 (m), 2619.82 (w), 1844.58 (w), 1714.41 (s), 1683.55 (s), 1646.91 (m), 1599.66 (m), 1562.06 (s), 1458.89 (w), 1433.82 (m), 1402.0 (w), 1343.18 (w), 1268.93 (m), 1240.97 (s), 1150.33 (w), 1112.73 (w), 995.08 (w), 963.26 (w), 913.12 (m), 761.74 (m), 729.92 (w), 674.96 (m), 540.93 (w), 500.43 (w), 419.44 (w).

#### 2.3.2. Bis(2,5-dicarboxyphenyl)adipoamide, H_4_**L**^2^

Typically, only one kind of solvent is used in the reflux process during the synthesis of the ligand, but here, two different solvents (Pyridine and DMF) were used to synthesize a modified version of H_4_**L**^2^. 10 mL pyridine solution of triethylamine (1.11 g, 10.94 mmol) was added slowly to the 15 mL pyridine solution of 2-amino-isophthalic acid (1.98 g, 10.94 mmol) kept in the reflux flask facilitated with the magnetic stirrer, and the mixture was stirred for 30 min. Adipoyl chloride (1.00 g, 5.47 mmol) in 15 mL DMF was carefully added dropwise to the stirring solution mixture, which was refluxed for one day. After cooling, the mixture was poured into 300 mL ether to induce the precipitation, and the solution was stirred for some hours. Finally, the solution was filtered, and the residue was washed with 3 × 300 mL ether to obtain the white powder. Yield: 2.51 g (97%). Anal Calcd for C_22_H_20_N_2_O_10_ (MW = 472.11): C, 55.93; H, 4.27; N, 5.93. Found: C, 55.48; H, 4.24; N, 6.02. ^1^H-NMR (DMSO-*d*_6_, 400 MHz, 25 °C): δ 1.68 (t, 4H; CH_2_), 2.43 (t, 4H; CH_2_), 7.64 (d, 1H; ArH), 8.02 (d, 1H; ArH), 9.00 (s, 1H; ArH), 11.06 (s, 2H; NH), Figure S4. ^13^C-NMR (DMSO-*d*_6_, 400 MHz, 25 °C): δ 171.72 (–CONH–), 169.35 (–COOH–C1), 167.00 (–COOH–C4), 140.98 (NH–terephthalate–C2), 135.63 (NH–terephthalate–C4), 131.69 (NH–terephthalate–C6), 123.45 (NH–terephthalate–C5), 121.38 (NH–terephthalate–C1), 120.71 (NH–isophthalate–C3), 37.51 (–CH_2_– bonded to amide), 24.71 (–CH_2_– aliphatic), Figure S5. IR (cm^−1^): 3773.05 (w), 3704.58 (w), 3439.42 (w), 2976.59 (m), 2938.98 (m), 3738.42 (m), 2676.71 (s), 2493.51 (m), 2359.48 (m), 1691.27 (s), 1580.38 (s), 1529.27 (s), 1475.28 (s), 1431.89 (s), 1398.14 (m), 1199.51 (s), 1036.55 (s), 907.34 (m), 805.13 (m), 758.85 (s), 512.97 (w), 404.98 (w). TOF, ESI-MS, methalnol (-): *m*/*z* 471.10[M − H]^−^, 493.09 [M + Na − 2H]^−^, Figure S6.

#### 2.3.3. {[Mg_2_(**L**^1^)(H_2_O)_2_]·2EtOH.3H_2_O}_n_, **1**

A mixture of Mg(NO_3_)_2_·6H_2_O (0.10 g, 0.40 mmol), H_4_**L**^1^ (0.047 g, 0.10 mmol) and a mixed solvent (EtOH:H_2_O = 5:1, 6 mL) were placed in a 23 mL Teflon-lined stainless container, which was bubbled with nitrogen gas, sealed and then heated at 120 °C for 72 h under autogenous pressure. Light yellow block crystals were collected, washed by ethanol and then dried under a vacuum. Yield: 0.054 g (89%). Anal Calcd for C_22_H_26_Mg_2_N_2_O_15_ (MW = 607.06) excluding lattice ethanol: C, 43.53; H, 4.32; N, 4.61. Found: C, 43.72; H, 4.39; N, 4.15. IR (cm^−1^): 3646.73 (w), 3101.94 (m, wide), 1680.66 (m), 1617.02 (m), 1557.24 (s), 1432.85 (s), 1372.10 (s), 1190.83 (m), 966.16 (w), 902.52 (m), 784.89 (m), 713.53 (m), 447.40 (m).

#### 2.3.4. [Mg_2_(**L**^1^)(H_2_O)_8_]_n_, **2**

The synthetic procedure for **2** is similar to that for **1**, except that the solvent system adopted is 4:1, 10 mL, instead of 5:1, 6 mL. Two types of crystals were obtained: block crystals **1** and light yellowish plate crystals **2**, which were manually separated under a microscope. The crystals were collected individually, washed with ethanol, and then dried under vacuum. Yield of **1**: 0.031 g, (51%). Yield of **2**: 0.024 g (36%). Anal Calcd for C_22_H_32_ Mg_2_N_2_O_18_ (MW = 661.10): C, 39.97; H, 4.88; N, 4.24. Found: C, 39.63; H, 4.91; N, 4.12. IR (cm^−1^): 3657.34 (w), 3527.17 (w), 3093.26 (m, wide), 1677.77 (s), 1546.63 (s), 1376.93 (s), 1270.86 (s), 1183.11 (m), 897.70 (m), 792.60 (s), 715.46 (s), 560.22 (m), 451.26 (m). 

#### 2.3.5. {[Mg_2_(**L**^2^)(H_2_O)_6_]·H_2_O}_n_, **3**

The synthetic procedure for **3** is similar to that for **2**, except that H_4_**L**^2^ (0.047 g, 0.10 mmol) is used instead of H_4_**L**^1^, without passing N_2_ gas to the solution. Heating temperature (120 °C) was changed to 140 °C for 48 h and the oven was cooled naturally. Colorless block crystals of **3** were collected, washed with ethanol, and then dried under vacuum. Yield: 0.055 g (86%). Anal Calcd for C_22_H_30_Mg_2_N_2_O_17_ (MW = 643.08): C, 41.09; H, 4.70; N, 4.36. Found: C, 41.25; H, 4.85; N, 4.05. IR (cm^−1^): 3674.69 (w), 2954.41 (w), 1650.77 (m), 1564.95 (s), 1511.92 (s), 1415.49 (s), 1375.00 (s), 1296.89 (m), 1251.58 (m), 1181.19 (m), 1083.80 (w), 964.22 (m), 780.06 (s), 693.28 (m), 514.90 (m), 432.94 (m), 418.48 (m), 404.98 (m).

### 2.4. X-ray Crystallography

The diffraction data for complexes of **1**–**3** were collected on a Bruker AXS SMART APEX II diffractometer at 296 K, which was equipped with a graphite-monochromated MoKα (Kα = 0.71073 Å) radiation. Data collection and reduction were performed by standard methods with use of well-established computational procedures [32,33]. Positions of some of the heavier atoms were located by the direct or Patterson method and the remaining atoms were found in a series of alternating difference Fourier maps and least-square refinements [34]. Basic information pertaining to crystal parameters and structure refinement is summarized in Table 1. Selected bond distances and angles are listed in Table S1.

## 3. Results and Discussion

### 3.1. Synthesis

The crystals of Complex **1** were obtained from the solvent system of 5 mL ethanol and 1 mL water (ratio of EtOH:H_2_O = 5:1, total volume 6 mL), whereas those of both **2** and **3** were obtained from 8 mL ethanol and 2 mL water (ratio of EtOH:H_2_O = 4:1, total volume 10 mL), keeping all other experimental condition the same in both cases. It is interesting to note that the subtle differences in ratio and volume of the solvent system are significant for the formation of the single crystals of **1** and **2**. Further, we have attempted to investigate the possibility of transformation between **1** to **2** with their mutual exchange of solvent system. However, no transformation between **1** and **2** can be observed, indicating that both **1** and **2** are hydrothermally stable products.

### 3.2. Structrual Descriptions

#### 3.2.1. Crystal Structure of **1**

**Figure 1:**
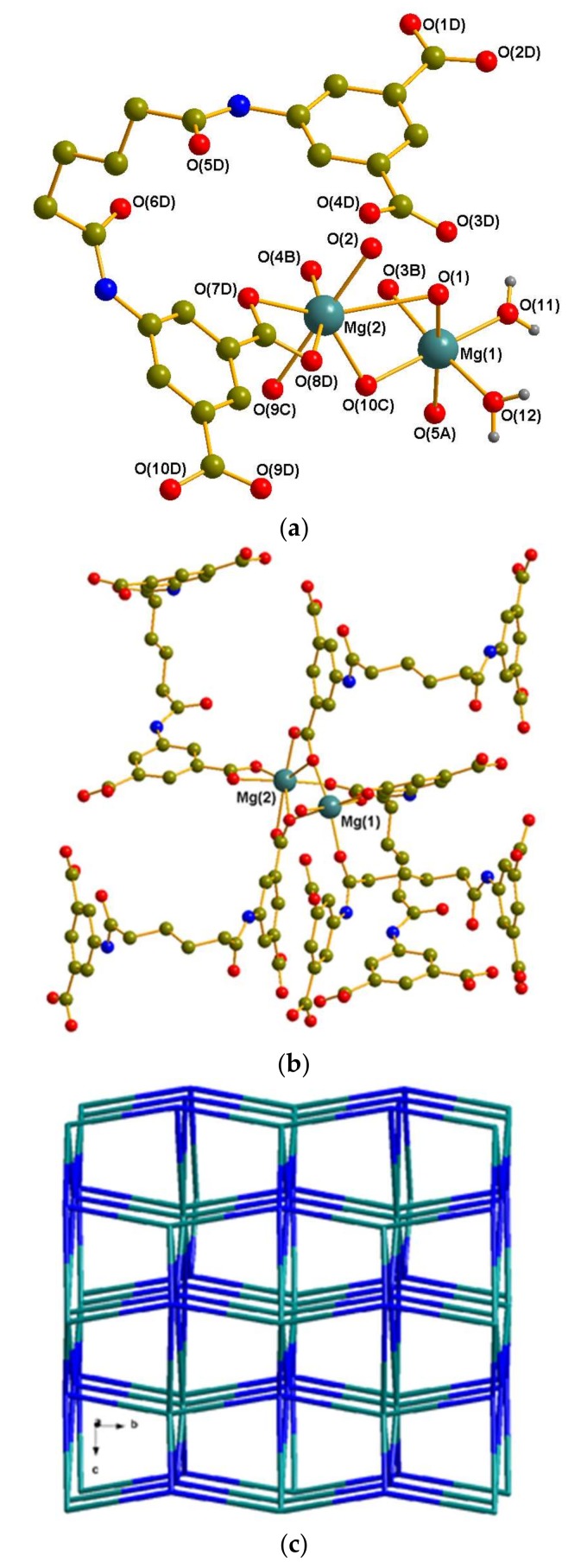
(**a**) Coordination environment of **1**; Symmetry Code: (A) −x + 3/2, y + 1/2, z + ½; (B) −x + 1, −y + 1, z + 1/2, (C) x, y + 1, z, (D) −x + 1, −y + 1, z − ½; (**b**) A view showing a dinuclear Mg_2_ unit as a 5-connected node; (**c**) A 3D (4^4^.6^6^)- pcu-5-Pmna topological net of **1**.

Complex **1** crystallizes in the orthorhombic space group *Pna*2_1_, and its asymmetric unit contains two Mg(II) ions, one (**L**^1^)^4−^ ligand, two bonded water, two lattice ethanol and three lattice water. Figure 1a depicts the coordination environment of the two Mg(II) metal centers. It can be shown that the Mg(1) center is linked by six oxygen atoms (two from water, three from carboxylate groups of three different ligands and one from the amide part of other ligand), resulting in a distorted octahedral geometry. The equatorial plane is formed by the coordination of O(3B), O(10C), O(11) and O(12) atoms, where the axial positions are occupied by O(1) and O(5A) atoms. Likewise, the Mg(2) metal center is coordinated by seven oxygen atoms (three pairs from the carboxylate groups of three different ligands and one from a carboxylate group of other ligand), forming a distorted pentagonal bipyramidal geometry. Here, the pentagonal plane is formed by the coordination of O(1), O(2), O(7D), O(9C) and O(10C) atoms while the axial positions are occupied by O(4B) and O(8D) atoms. Thus, two Mg(II) centers are bridged and chelated by five different carboxylate ligands forming a dinuclear Mg(II) unit as a 5-connected node, Figure 1b. The Mg-O distances range from 1.996(3) of Mg1-O4B to 2.725(3) Å of Mg2-O1, while Mg…Mg distance is 3.3424(19) Å. Notably, each tetracarboxylate ligand connects eight Mg(II) ions through nine oxygen atoms to form a 3D net. Topologically, both the Mg_2_ and (**L^1^**)^4−^ can be regarded as 5-connected nodes and afford a uninodal 5-connected net with the non-interpenetrating (4^4^.6^6^)-pcu-5-Pmna topology, Figure 1c, determined using ToposPro [35]. While several 3-fold interpenetrated CPs that show pcu-5-Pmna topology can be found, only two with non-interpenetrating modes have been reported, as shown in the TTD database of ToposPro. A PLATON calculation [36] indicates 30.5% accessible free voids for **1** after the removal of guest molecules.

#### 3.2.2. Crystal Structure of **2**

**Figure 2:**
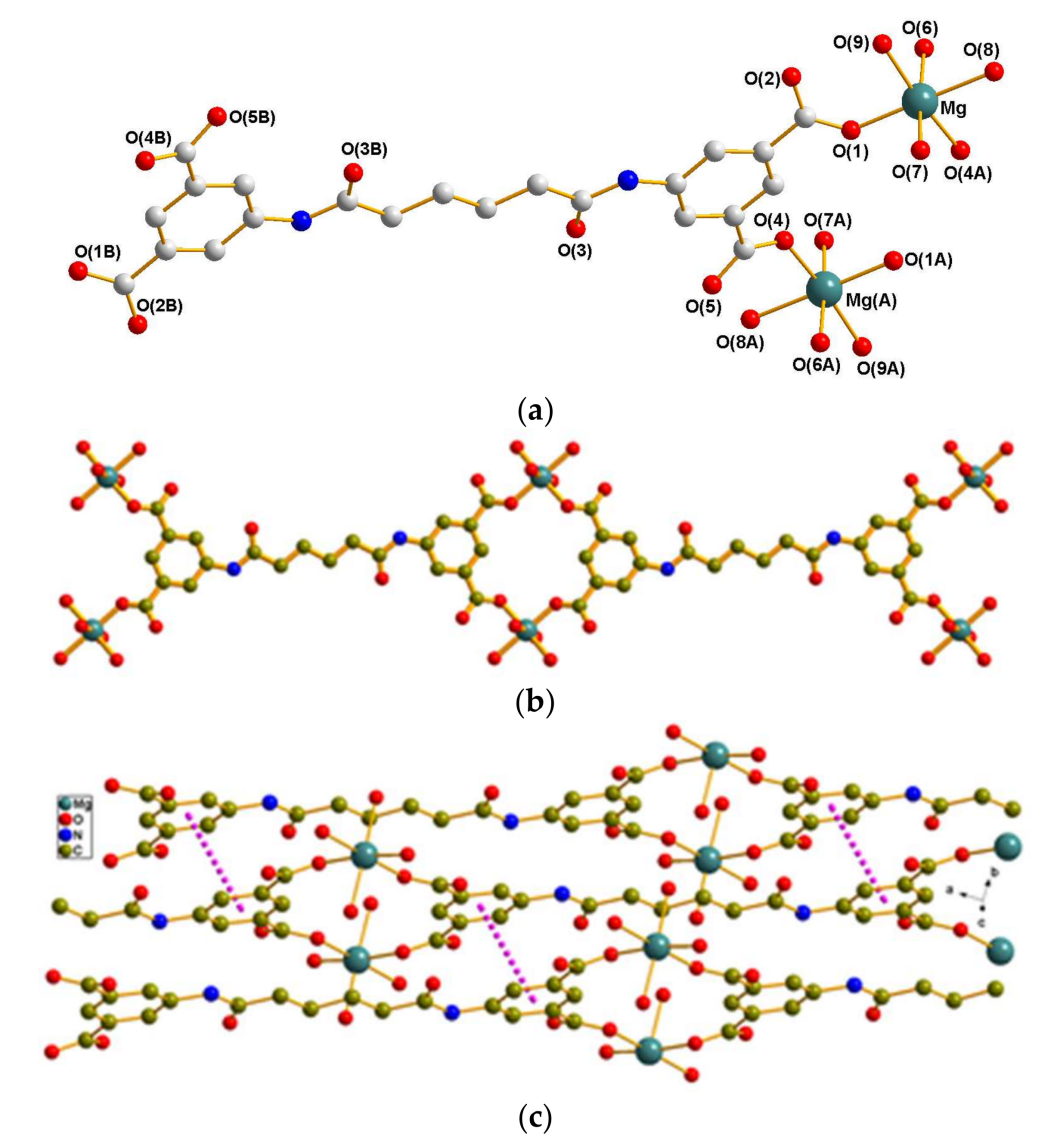
(**a**) Coordination environment of **2**; Symmetry Code: (A) -x, -y, -z. (**b**) A view showing the 1D structure of **2**. (**c**) A drawing showing π-π interaction in **2**, resulting a 2D sheet.

Single-crystal X-ray diffraction analysis reveals that Complex **2** crystallizes in the monoclinic space group *P*2_1_/n and its asymmetric unit contains one Mg(II) ion, half (**L**^1^)^4−^ ligand and four bonded water molecules. Figure 2a shows the arrangement around the Mg(II) metal center, which is six-coordinated by two oxygen atoms of the carboxylate groups of the different ligands and four oxygen atoms of the water molecules, resulting in a distorted octahedral geometry. The equatorial plane is formed by the coordination of O(1), O(4A), O(8) and O(9) atoms, where the axial positions are occupied by O(6) and O(7) atoms. In Complex **2**, each of the four carboxylate groups of the (**L**^1^)^4−^ ligand coordinates to one Mg(II) ion through one of the two oxygen atoms, which is repeated in the linear fashion to form a 1D chain, Figure 2b. Another notable feature is that the 1D linear planar structures of **2** interact with each other through the π-π interaction (3.68 Å), forming a 2D supramolecular structure, Figure 2c. In Complex **2**, the Mg-O distances range from 2.0456(10)–2.1864(12) Å while Mg…Mg distance is 7.8612(1) Å. 

#### 3.2.3. Crystal Structure of **3**

**Figure 3:**
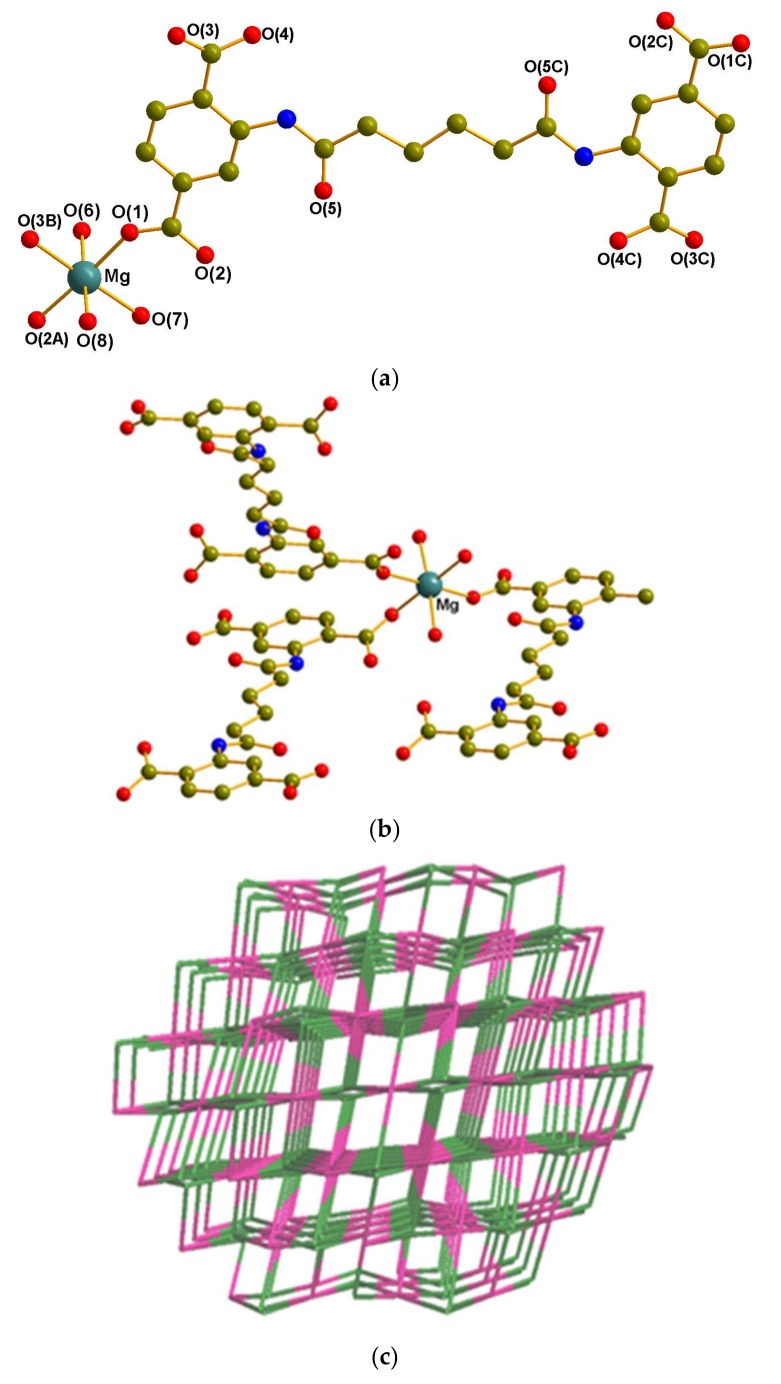
(**a**) Coordination environment of **3**; Symmetry Code: (A) −x, y − 1/2, −z + ½, (B) −x + 1, y − 1/2, −z + ½; (**b**) A view showing a Mg(II) unit as a 3-connected node; (**c**) 3D {4.6^2^}_2_{4^2^.6^10^.8^3^}-***rtl*** topological net of **3**.

Single-crystal X-ray diffraction studies reveal that **3** crystallizes in the monoclinic space group *P*2_1_/c, featuring a 3D framework. Its asymmetric unit contains one Mg(II) ion, half (**L**^2^)^4−^ ligand, three coordinated H_2_O molecules and one crystallized water molecule. As shown in Figure 3a, the Mg(II) ion is in the distorted octahedral fashion, coordinated by six oxygen atoms, with three from the carboxylate groups of three different (**L**^2^)^4−^ ligands and three from water molecules. The equatorial plane is formed by the coordination of O(1), O(3B), O(2A) and O(7) atoms where the axial positions are occupied by O(6) and O(8) atoms. Each Mg(II) center behaves as a three-connected single node, Figure 3b, while two Mg(II) centers are bridged and coordinated by five different carboxylate ligands forming a dinuclear Mg_2_ unit as a 5-connected node, Figure S7. In Complex **3**, each (**L**^2^)^4−^ ligand connects six Mg(II) ions through six oxygen atoms, forming a six-connected node. Thus, both Mg(II) and (**L**^2^)^4−^ act as 3- and 6-connected nodes, respectively, to afford the 3,6-connected 2-nodal ***rtl*** rutile type 3D net having the point symbol of {4.6^2^}_2_{4^2^.6^10^.8^3^}, Figure 3c. It is noted that the Mg-O distances range from 2.0141(19)–2.130(2) Å, while the Mg…Mg distance is 4.8973(1) Å.

### 3.3. Coordination Modes and Ligand Conformation

**Figure 4:**
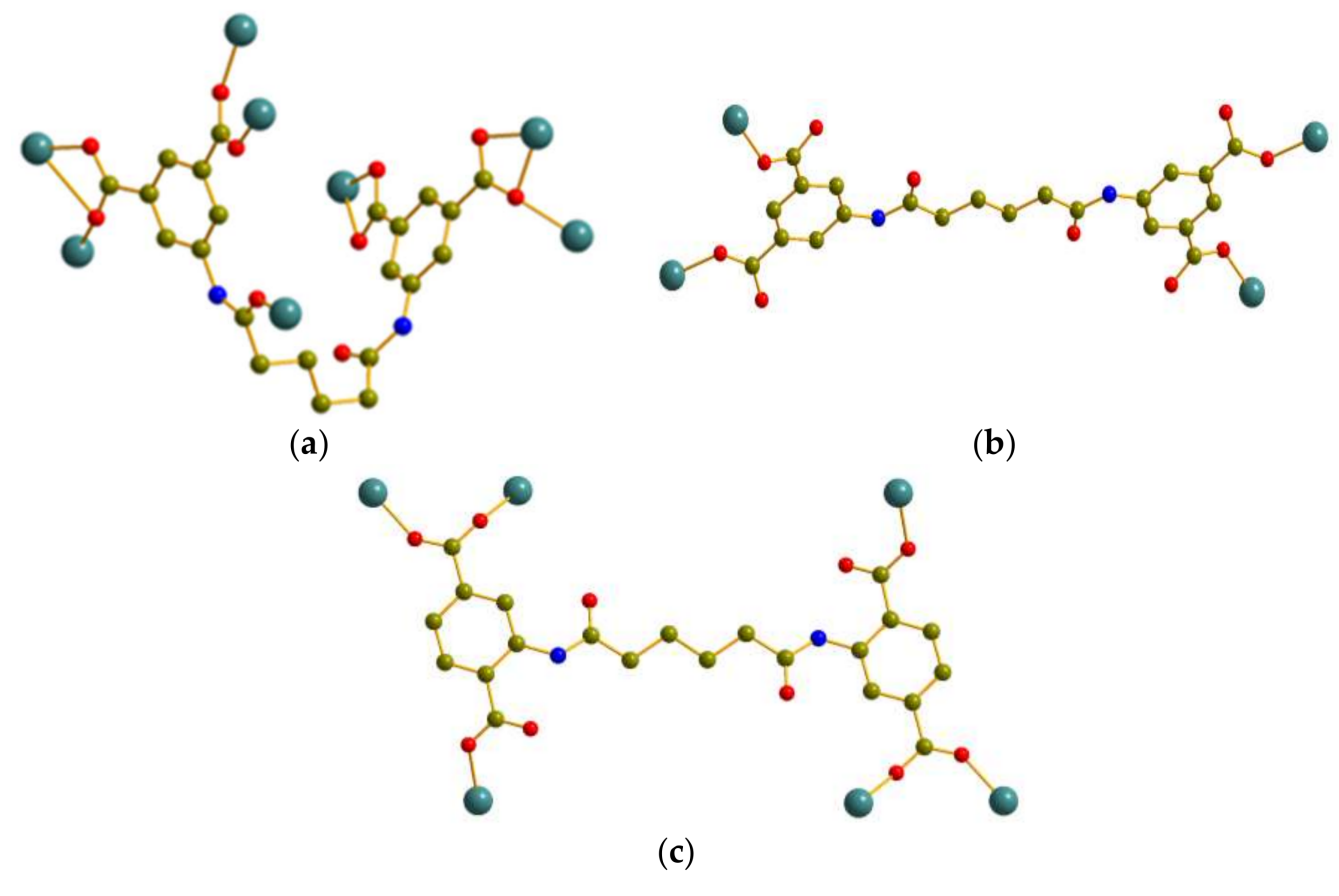
Various coordination modes of tetracarboxylate ligands: (**a**) (**L**^1^)^4−^ in **1**, (**b**) (**L**^1^)^4−^ in **2**, (**c**) (**L**^2^)^4−^ in **3**.

From Figure 4, it is clear that the tetracarboxylate ligands (**L**^1^)^4−^ and (**L**^2^)^4−^ exhibit various coordination modes with Mg(II) metal centers. The (**L**^1^)^4−^ ligands show drastically different bonding modes in **1** and **2**. More interestingly, the (**L**^1^)^4−^ ligand in **1** bridges eight Mg(II) ions through nine oxygen atoms, which is participated in by four different coordination modes from carboxylate groups and carbonyl oxygen atoms, including two chelating/bridging (carboxylate group), one bridging (carboxylate group), one chelating (carboxylate group) and one monodentate coordination (carbonyl oxygen atom). Such a bonding mode is unique for the tetracarboxylate ligands as linkers in the CPs, presumably due to the existence of the amide carbonyl oxygen atoms in the (**L**^1^)^4−^ ligand. Likewise, the (**L**^1^)^4−^ ligand in **2** connect four Mg(II) ions through four monodentate carboxylate oxygen atoms, and the (**L**^2^)^4−^ ligand in **3** link six Mg(II) ions through two bridging carboxylate groups and two monodentate carboxylate oxygen atoms. On the other hand, the semi-rigid ligand DBIP^4−^ [H_4_DBIP = 5-(3,5-dicarboxybenzyloxy)-isophthalic acid] in [Mg_2_(DBIP)(H_2_O)_4_(μ_2_-H_2_O)]·6H_2_O links six Mg(II) ions through two bridging carboxylate groups and two monodentate carboxylate oxygen atoms, while that in [Cd_2_(DBIP)(H_2_O)_2_(DMA)]·5DMA·5H_2_O connect eight Cd(II) ions through four bridging carboxylate groups [20].

The A and G conformations are symbolized when the C-C-C-C torsion angle (θ) is 180 ≥ θ > 90° and 0 ≤ θ ≤ 90°, respectively. Based on this descriptor [7], the ligands (**L**^1^)^4−^ and (**L**^2^)^4−^ can be arranged in *anti*-*anti*-*anti* (AAA), *anti*-*anti*-*gauche* (AAG), *anti*-*gauche*-*anti* (AGA), *anti*-*gauche*-*gauche* (AGG), *gauche*-*anti*-*gauche* (GAG) and *gauche*-*gauche*-*gauche* (GGG) conformations. Accordingly, the (**L**^1^)^4−^ ligand of **1** can be assigned as AAG while both (**L**^1^)^4−^ of **2** and (**L**^2^)^4−^ of **3** as AAA. Likewise, each conformation can adopt *cis* or *trans* arrangements based on the relative orientation of the C=O (or N-H) groups. Accordingly, both ligands in **1**–**3** can be assigned as *trans*. Hence, the conformations of both ligands, as a whole, can be assigned as AAG *trans* conformation in **1**, while AAA *trans* conformation in **2** and **3**. These ligand conformations also differ in the dihedral angle between the two phenyl rings, i.e., the two rings are coplanar or twisted about the C-N bonds. The dihedral angles between the two phenyl rings of (**L**^1^)^4−^ ligands in **1** is 34.7° while for the case of **2** and **3**, the pair of phenyl rings of the ligands is coplanar, i.e., 0°.

### 3.4. PXRD Patterns and Thermogravimetric Analysis

To identify the bulk purities of **1**–**3**, the experimental pattern of powder X-ray diffraction (PXRD) is compared with the simulated pattern obtained from the single-crystal X-ray data. As shown in Figures S8–S10, the experimental patterns match quite well with their corresponding simulated ones, which demonstrates that complexes **1**–**3** show bulk purities. Figure S11 represents the TGA curves of Complexes **1**–**3**. From the TGA curve of **1**, it is clear that the first wt. loss in 25–92 °C is due to the removal of lattice ethanol (obsd. 12.5%, calcd. 13.17%), followed by the 2nd wt. loss of lattice water (obsd. 7.68%, calcd. 7.73%) at 93–155 °C. In the temperature range from 155 to 413 °C, the bonded water molecules were completely lost; and finally, the (**L**^1^)^4−^ ligand started to decompose at temperatures above 413 °C. The TGA curve of **2** shows a weight reduction of 21.33% (calcd. 21.8%) in the temperature range from 25 to 190 °C, corresponding to the release of bonded water and, after that, the residual framework maintains a long stable period up to 420 °C, above which the entire skeleton of **2** deteriorates. Compound **3** exhibits a slight weight reduction of 3.23% (calcd. 2.79%) between 25 and 74 °C due to the loss of lattice water, followed by the 2nd weight loss of bonded water of 17.05% (calcd. 16.79%) between 75 and 187 °C; and above 378 °C, the complex started to decay. Comparatively, the dehydrated phase of **2** is more stable than the others up to approximately 420 °C.

### 3.5. Structural Transformation

**Figure 5:**
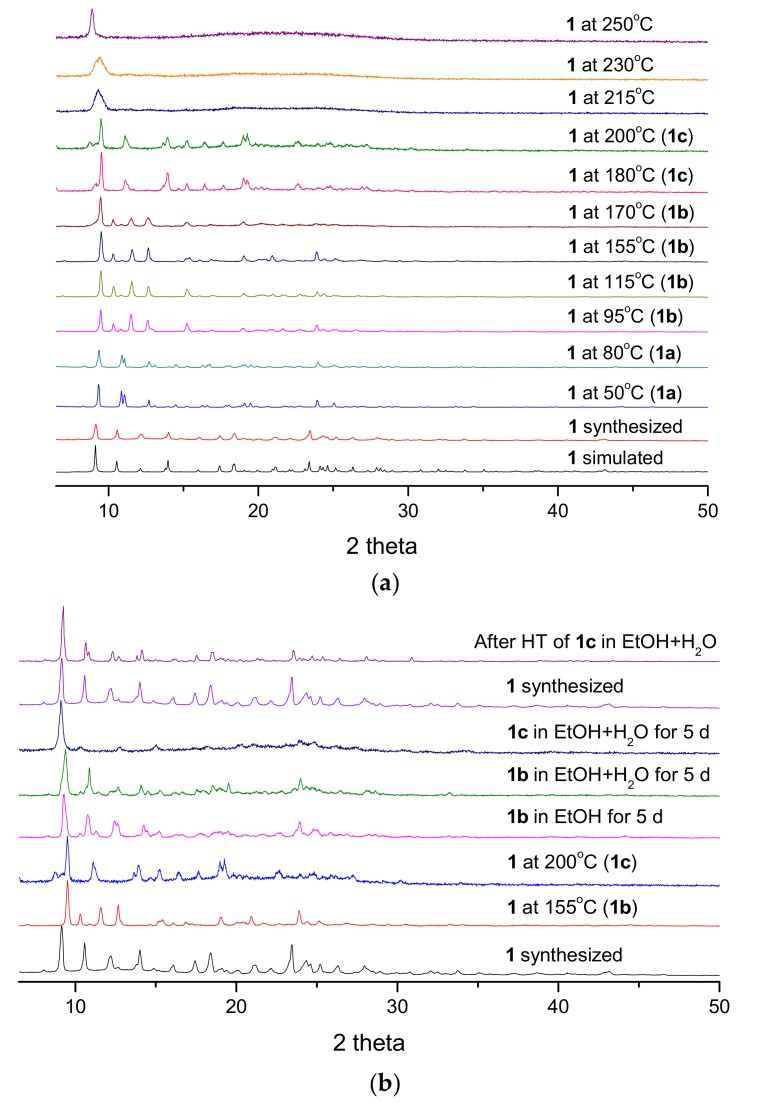
(**a**) Variable temperature powder XRD patterns of **1** showing a series of stepwise structural transformations from **1** to **1a** to **1b** to **1c**; (**b**) Powder XRD patterns showing reversible structural transformation from **1b** or **1c** to **1** by applying various methods.

After verification of the phase purity of Complex **1**, we heated the powder of **1** for 4 h under vacuum at various temperatures from 50 to 250 °C to observe the thermal stability, Figure 5a. Firstly, the powder of **1** was activated by heating at 50 °C and then 80 °C. The powder patterns at both temperatures were found to be similar, but were different from that of the original synthesized **1**, indicating the first-step structural transformation from **1** to **1a**. Similarly, powder **1a** was further heated successively at 95, 115, 155 and then 170 °C. The PXRD patterns at these temperatures were about the same, but were typically different from those of **1a** or **1**, representing the second-step structural transformation from **1a** to **1b**. Furthermore, third-step structural transformation from **1b** to **1c** was also noticed when the powder of **1b** was heated successively at 180 °C and then 200 °C. When powder **1c** was further activated at 215, 230 and 250 °C, we observed only one peak shifted slightly toward the left, which may represent the collapse of the framework. To monitor whether the activated samples can be reversed to the original one or not, the activated sample **1b** (at 155 °C) was immersed in ethanol or the mixture of ethanol and water for 5 days, Figure 5b. The powder patterns obtained from the both solvent systems look nearly identical to the original synthesized **1**, which indicate the reversible structural transformation from **1b** to **1**. Comparatively, the PXRD pattern obtained from the mixed solvent system was slightly better than that obtained from ethanol only. Likewise, the powder pattern obtained after immersing **1c** (at 200 °C) in the same solvent mixture for 5 days seemed nearly identical to the synthesized **1**, indicating the possibility of a reversible structural transformation from **1c** to **1**. Likewise, activated **1c** can also be transformed into original **1** under hydrothermal conditions by employing the same solvent ratio as for **1**. Overall, from the variable temperature powder patterns of **1**, it can be shown that the structure of Complex **1** shows a series of stepwise structural transformations from **1** to **1a** to **1b** and to **1c** on heating, and nearly reversible structural transformation from **1b** and **1c** to **1** on immersing the activated samples in solvent mixtures or by the hydrothermal process.

**Figure 6:**
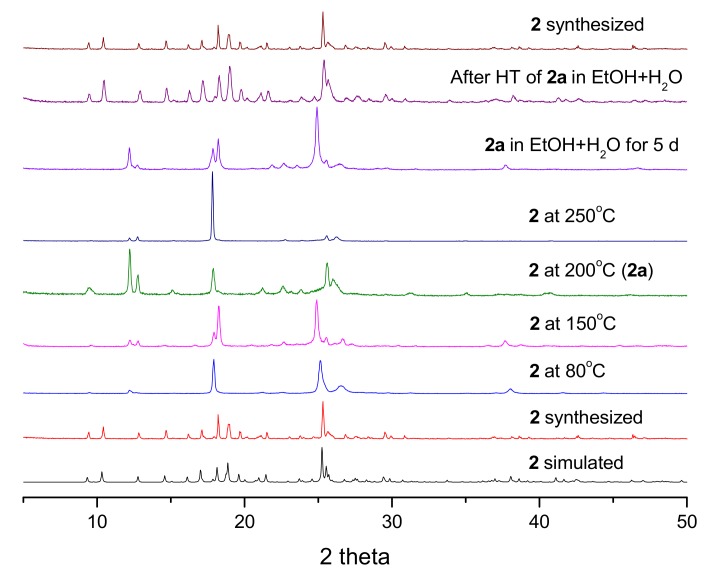
Variable temperature powder XRD patterns of **2** showing reversible structural transformation.

Figure 6 indicates that Complex **2** displays minor changes in PXRD patterns on heating at 80 and 150 °C, while a new pattern forms at 200 °C (designated as a dehydrated product **2a**), which remains similar up to 250 °C. Since activated **2a** is water soluble, we immersed **2a** in the mixture of ethanol and traces of water for 5 days, but the pattern remained almost the same, with only minor changes. On the other hand, activated **2a** under hydrothermal conditions could be converted into the original **2**, showing a reversible structural transformation. The PXRD pattern started to change on heating the powder of **3** at 80 °C, and completely changed at 190 °C, with the result being designated as the dehydrated product **3a**, Figure S12. No reversible transformation from **3a** to **3** was observed, even though various methods were carried out. The differences in the donor atom positions between the isomeric tetracarboxylate ligands (**L**^1^)^4−^ and (**L**^2^)^4−^ may be responsible for the reversibility of the structural transformations in **1**–**3**.

### 3.6. Absorption and Luminescence Properties

**Figure 7:**
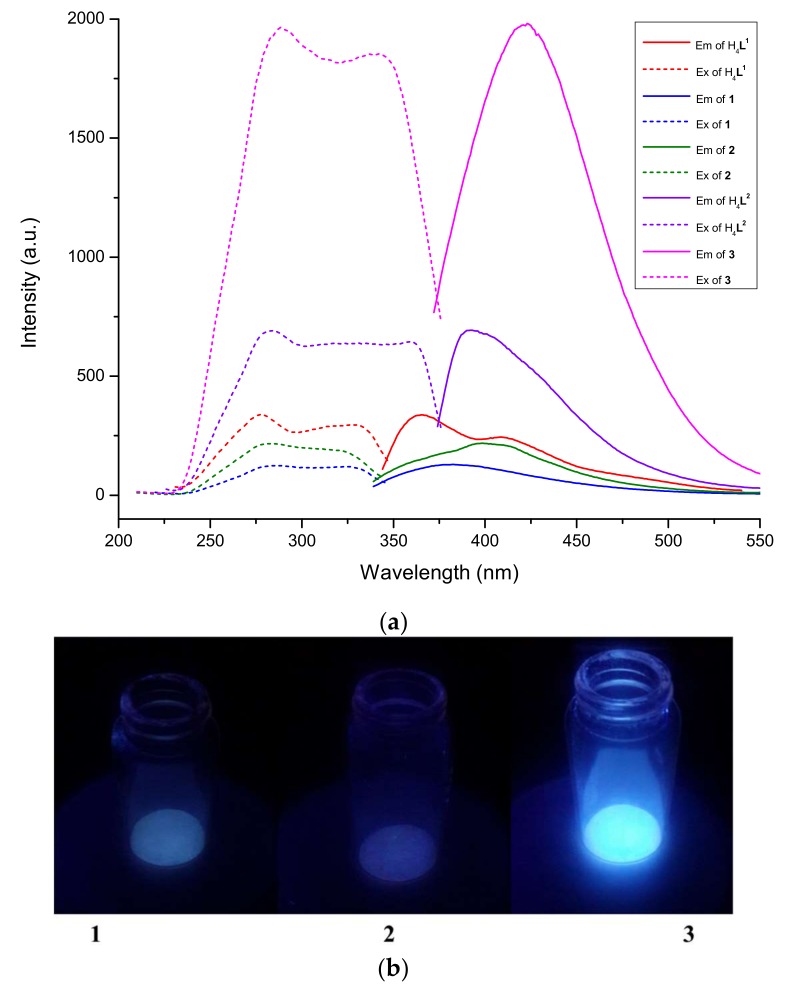
Emission and excitation spectra of (**a**) H_4_**L**^1^, **1**, **2**, H_4_**L**^2^ and **3**; (**b**) Images of powder **1**–**3** under a UV lamp at 365 nm in a dark room.

Not only the d^10^ metal centers, but also the Mg(II) CPs have the potential ability to quench, shift and enhance the emission wavelength of organic ligands through the coordination of metal centers, and therefore, they may also be regarded as promising candidates for prospective luminescent applications as light-emitting diodes (LEDs), probes and sensors [37,38,39,40,41]. Figure S13 depicts the solid-state UV/vis spectra of **1**–**3** and ligands (H_4_**L**^1^ and H_4_**L**^2^), which display sharp absorption bands ranging from 200 to 400 nm. Likewise, the solid-state emission spectra for all complexes and ligands (using equimolar quantities) were measured at room temperature under identical experimental conditions, as shown in Table 2 and Figure 7a. The free ligands H_4_**L**^1^ and H_4_**L**^2^ exhibit emission peaks at 365 nm (with a shoulder peak at 408 nm) and 391 nm upon excitation at 278 nm and 284 nm, respectively, in the solid state, while emissions at 353 nm (in EtOH) and 351 nm (in DMF) for H_4_**L**^1^ and 389 nm (in EtOH) and 388 nm (in DMF) are observed for H_4_**L**^2^, Figures S14 and S15. Since the emission wavelengths of both ligands in solution and solid state are nearly similar, the effect of intermolecular interactions is negligible, and they may be tentatively ascribed to the intraligand n⟶π* or π⟶π* transitions [41].

In addition, **1** and **2** show broad emission bands at 384 nm and 399 nm upon excitations at 287 nm and 283 nm, respectively, while **3** displays an emission maximum at 423 nm upon excitation at 289 nm. By comparing the emission spectra of **1**–**3** and ligands, all of the complexes exhibit red shifts (19 nm for **1**, 34 nm for **2** and 32 nm for **3**) with respect to their corresponding free ligands, which may be attributed to the increase of the ligand conjugation degree after coordination with Mg^2+^ ions [42,43]. Since Mg(II) ions, having 1s^2^2s^2^2p^6^ electronic configuration, are difficult to oxidize or reduce in nature, the emission is most probably due to the ligand-centered n⟶π* or π⟶π* transition. Furthermore, the images of powders of **1**–**3** under UV lamp at 365 nm, Figure 7b, indicate that Complex **3** emits the more intense blue light than **1** and **2**. Based on this observation, the (**L**^2^**)**^4−^ in **3** can be assumed to be more rigid [22] than (**L**^1^)^4−^ in **1** and **2**, which can also be ascribed to the different ligand isomerism, although both of them have the same -(CH_2_)_4_- spacer.

## 4. Conclusions

We have successfully synthesized a new tetracarboxylic acid H_4_**L**^1^, and its isomer H_4_**L**^2^ has also been prepared in a modified version. The reactions of H_4_**L**^1^ with Mg(II) salt under hydrothermal conditions gave **1** and **2**, which possess a 3D net with a rare non-interpenetrating (4^4^.6^6^)-pcu-5-Pmna topology and a 1D chain, respectively. Here, the ratio and volume of the solvent system play vital roles in the formation of the crystals of **1** and **2**. On the other hand, the solvothermal treatment of H_4_**L**^2^ with Mg(II) salt produces Complex **3**, which has a *rutile*-type 3D framework. Complexes **1**–**3** can be regarded as unique examples of Mg(II) CPs from flexible isomeric tetracarboxylate ligands. The different structures of **1**–**3** are presumably due to the structure-directing effect of isomeric ligands and the different experimental conditions. Notably, the tetracarboxylate ligand in **1** connects eight Mg(II) ions through nine oxygen atoms, whereas those of **2** and **3** link four Mg(II) ions through four oxygen atoms and six Mg(II) ions through six oxygen atoms, respectively. In addition, Complex **1** displays a stepwise structural transformation on heating, and the activated product can be reversibly transformed to the original **1** on hydrothermal or immersion in the solvent system. While such reversible structural transformations were also observed in **2**, Complex **3** exhibited an irreversible structural transformation. The reversible and irreversible transformations in **1**–**3** can most probably be ascribed to the different donor positions between the isomeric (**L**^1^)^4−^ and (**L**^2^)^4−^ ligands. Furthermore, all three complexes exhibited visible blue light emissions under UV light, with that of Complex **3** being found much more intense.

## Figures and Tables

**Table 1:** Crystal data for Complexes **1**–**3**.

Compound	1	2	3
Formula	C_26_H_38_Mg_2_N_2_O_17_	C_22_H_32_Mg_2_N_2_O_18_	C_22_H_30_Mg_2_N_2_O_17_
Fw	699.20	661.11	643.10
crystal system	Orthorhombic	Monoclinic	Monoclinic
space group	*Pna*2_1_	*P*2_1_/*n*	*P*2_1_/*c*
a, Å	16.7546 (2)	10.4098 (2)	10.1719 (2)
b, Å	14.7326 (2)	7.4955 (1)	9.5855 (2)
c, Å	12.8347 (2)	18.2183 (3)	15.4358 (3)
α,°	90	90	90
β,°	90	96.671 (1)	105.778 (1)
γ,°	90	90	90
V, Å^3^	3168.10 (8)	1411.89 (4)	1448.33 (5)
Z	4	2	2
Dcalc, g/cm^3^	1.466	1.555	1.475
μ(Mo Kα), mm^−1^	0.157	0.174	0.165
Range (2θ) for data collection, deg	1.841 ≤ 2θ ≤ 28.314	2.152 ≤ 2θ ≤ 28.294	2.742 ≤ 2θ ≤ 26.00
independent reflections	7742 [R (int) = 0.0433]	3490 [R (int) = 0.0162]	2845 [R (int) = 0.0314]
data/restraints/parameters	7742/9/436	3490/0/223	2845/0/198
quality-of-fit indicator c	1.057	1.059	1.047
final R indices [I > 2σ(I)] a, b	R1 = 0.0486, wR2 = 0.1242,	R1 = 0.0399, wR2 = 0.1125	R1 = 0.0520, wR2 = 0.1456
R indices (all data)	R1 = 0.0671, wR2 = 0.1366	R1 = 0.0425, wR2 = 0.1148	R1 = 0.0670, wR2 = 0.1586

^a^ R_1_ = Σ||F_o_| − |F_c_||/Σ|F_o_|; ^b^ wR_2_ = [Σw(F_o_^2^ − F_c_^2^)^2^/Σw(F_o_^2^)^2^]^1/2^. w = 1/[σ^2^(F_o_^2^) + (ap)^2^ + (bp)], p = [max(F_o_^2^ or 0) + 2(F_c_^2^)]/3. a = 0.0825, b = 0.00, **1**; a = 0.0633, b = 0.72, **2**; a = 0.0867, b = 1.10, **3**; ^c^ quality-of-fit = [Σw(|F_o_^2^| − |F_c_^2^|)^2^]/[N_observed_ − N_parameters_].

**Table 2:** Solid-state PL of Ligands (H_4_**L**^1^ and H_4_**L**^2^) and Complexes **1**–**3**.

Compound	Excitation λ_(ex)_ nm	Emission λ_(em)_ nm
H_4_**L**^1^	278, 316, 328	365, 408
**1**	287, 324	384
**2**	283	399
H_4_**L**^2^	284, 331, 369	391
**3**	289, 342	423

## Supplementary Materials

The following are available online at http://www.mdpi.com/2073-4360/10/4/371/s1. ^1^H-NMR (Figure S1), ^13^C-NMR (Figure S2) and ESI-MS (Figure S3) of H_4_**L**^1^, ^1^H-NMR (Figure S4), ^13^C-NMR (Figure S5) and ESI-MS (Figure S6) of H_4_**L**^2^, dinuclear Mg_2_ unit as a 5-connected node in **3**, (Figure S7) powder XRD patterns of **1**–**3** (Figures S8–S10), TGA curves of **1**–**3** (Figure S11), Variable temperature powder XRD of **3**, Figure S12, solid-state UV/Vis spectra of **1**–**3** (Figure S13), solution state Em/Ex of H_4_**L**^1^ (Figure S14), H_4_**L**^2^ (Figure S15), Crystallographic data for **1**–**3** have been deposited with the Cambridge Crystallographic Data Centre, CCDC No. 1520420–1520422.
