# Supplementary material for: Mg(II) Coordination Polymers Based on Flexible Isomeric Tetracarboxylate Ligands: Syntheses, Structures, Structural Transformation and Luminescent Properties
Source: Polymers (Basel). 2018 Mar 26;10(4):371. doi: 10.3390/polym10040371 (PMC6415218; doi:10.3390/polym10040371)

checkCIF/PLATON report

Structure factors have been supplied for datablock(s) 1, 2, 3

THIS REPORT IS FOR GUIDANCE ONLY. IF USED AS PART OF A REVIEW PROCEDURE FOR PUBLICATION, IT SHOULD NOT REPLACE THE EXPERTISE OF AN EXPERIENCED CRYSTALLOGRAPHIC REFEREE.

No syntax errors found. CIF dictionary Interpreting this report

Datablock: 1

Bond precision:	C-C = 0.0060 A		Wavelength=0.71073
Cell:	a=16.7546(2)	b=14.7326(2)	c=12.8347(2)
	alpha=90	beta=90	gamma=90
Temperature:	296 K		

	Calculated	Reported
Volume	3168.10(8)	3168.10(8)
Space group	P n a 21	P n a 21
Hall group	P 2c -2n	P 2c -2n
Moiety formula	C22 H20 Mg2 N2 O12, 2(C2 H6 O), 3(H2 O)	?
Sum formula	C26 H38 Mg2 N2 O17	C26 H38 Mg2 N2 O17
Mr	699.20	699.20
Dx,g cm-3	1.466	1.466
Z	4	4
Mu (mm-1)	0.157	0.157
F000	1472.0	1472.0
F000’	1473.22	
h,k,lmax	22,19,17	22,19,17
Nref	7884[4114]	7742
Tmin,Tmax	0.963,0.984	0.940,0.984
Tmin’	0.939	

Correction method= # Reported T Limits: Tmin=0.940 Tmax=0.984
AbsCorr = MULTI-SCAN

Data completeness= 1.88/0.98 Theta(max)= 28.314

R(reflections)= 0.0486(6175) wR2(reflections)= 0.1366(7742)

S = 1.057 Npar= 436

```
The following ALERTS were generated. Each ALERT has the format
       test-name_ALERT_alert-type_alert-level.
Click on the hyperlinks for more details of the test.
```

Alert level C

```
PLAT241_ALERT_2_C High   'MainMol' Ueq as Compared to Neighbors of         O1 Check
PLAT242_ALERT_2_C Low    'MainMol' Ueq as Compared to Neighbors of        Mg1 Check
PLAT242_ALERT_2_C Low    'MainMol' Ueq as Compared to Neighbors of        Mg2 Check
PLAT243_ALERT_4_C High   'Solvent' Ueq as Compared to Neighbors of        C25 Check
PLAT340_ALERT_3_C Low Bond Precision on  C-C Bonds ...............    0.00604 Ang.
PLAT355_ALERT_3_C Long    O-H (X0.82,N0.98A)  O13    -   H13C   ..       1.05 Ang.
```

Alert level G

```
PLAT002_ALERT_2_G Number of Distance or Angle Restraints on AtSite         12 Note
PLAT004_ALERT_5_G Polymeric Structure Found with Maximum Dimension          3 Info
PLAT007_ALERT_5_G Number of Unrefined Donor-H Atoms ..............         10 Report
PLAT066_ALERT_1_G Predicted and Reported Tmin&Tmax Range Identical          ? Check
PLAT172_ALERT_4_G The CIF-Embedded .res File Contains DFIX Records          5 Report
PLAT764_ALERT_4_G Overcomplete CIF Bond List Detected (Rep/Expd) .       1.22 Ratio
PLAT790_ALERT_4_G Centre of Gravity not Within Unit Cell: Resd.  #          3 Note
              C2 H6 O
PLAT792_ALERT_1_G The Model has Chirality at C21     (Polar  SPGR)          S Verify
PLAT860_ALERT_3_G Number of Least-Squares Restraints .............          9 Note
PLAT912_ALERT_4_G Missing # of FCF Reflections Above STh/L=  0.600         41 Note
PLAT978_ALERT_2_G Number C-C Bonds with Positive Residual Density           3 Note
```

```
   0 ALERT level A = Most likely a serious problem - resolve or explain
   0 ALERT level B = A potentially serious problem, consider carefully
   6 ALERT level C = Check. Ensure it is not caused by an omission or oversight
  11 ALERT level G = General information/check it is not something unexpected

   2 ALERT type 1 CIF construction/syntax error, inconsistent or missing data
   5 ALERT type 2 Indicator that the structure model may be wrong or deficient
   3 ALERT type 3 Indicator that the structure quality may be low
   5 ALERT type 4 Improvement, methodology, query or suggestion
   2 ALERT type 5 Informative message, check
```

Datablock: 2

Bond precision:	C-C = 0.0018 A		Wavelength=0.71073
Cell:	a=10.4098(2)	b=7.4955(1)	c=18.2183(3)
	alpha=90	beta=96.671(1)	gamma=90
Temperature:	296 K		

	Calculated	Reported
Volume	1411.89(4)	1411.89(4)
Space group	P 21/n	P 21/n
Hall group	-P 2yn	-P 2yn
Moiety formula	C11 H16 Mg N O9	?
Sum formula	C11 H16 Mg N O9	C22 H32 Mg2 N2 O18
Mr	330.56	661.11
Dx,g cm-3	1.555	1.555
Z	4	2
Mu (mm-1)	0.174	0.174
F000	692.0	692.0
F000’	692.61	
h,k,lmax	13,9,24	13,9,24
Nref	3497	3490
Tmin,Tmax	0.959,0.983	0.602,0.746
Tmin’	0.949	

Correction method= # Reported T Limits: Tmin=0.602 Tmax=0.746
AbsCorr = MULTI_SCAN

Data completeness= 0.998   Theta(max)= 28.294

R(reflections)= 0.0399(3222)   wR2(reflections)= 0.1148(3490)

S = 1.059   Npar= 223

The following ALERTS were generated. Each ALERT has the format
test-name_ALERT_alert-type_alert-level.
Click on the hyperlinks for more details of the test.

Alert level C

```
PLAT250_ALERT_2_C Large U3/U1 Ratio for Average U(i,j) Tensor ....        2.1 Note
PLAT911_ALERT_3_C Missing # FCF Refl Between THmin & STh/L=  0.600          3 Report
PLAT975_ALERT_2_C Check Calcd Residual Density  0.90A From      O6       0.40 eA-3
PLAT976_ALERT_2_C Check Calcd Residual Density  0.88A From      O6      -0.61 eA-3
PLAT977_ALERT_2_C Check the Negative Difference Density on     H6C      -0.61 eA-3
```

Alert level G

```
PLAT004_ALERT_5_G Polymeric Structure Found with Maximum Dimension          1 Info
PLAT007_ALERT_5_G Number of Unrefined Donor-H Atoms ..............          3 Report
PLAT045_ALERT_1_G Calculated and Reported Z Differ by a Factor ...       2.00 Check
PLAT232_ALERT_2_G Hirshfeld Test Diff (M-X)  Mg     --  O9      ..        8.0 s.u.
PLAT912_ALERT_4_G Missing # of FCF Reflections Above STh/L=  0.600          5 Note
PLAT933_ALERT_2_G Number of OMIT records in Embedded RES .........          1 Note
PLAT978_ALERT_2_G Number C-C Bonds with Positive Residual Density           9 Note
```

---

0 **ALERT level A** = Most likely a serious problem - resolve or explain
0 **ALERT level B** = A potentially serious problem, consider carefully
5 **ALERT level C** = Check. Ensure it is not caused by an omission or oversight
7 **ALERT level G** = General information/check it is not something unexpected

```
   1 ALERT type 1 CIF construction/syntax error, inconsistent or missing data
   7 ALERT type 2 Indicator that the structure model may be wrong or deficient
   1 ALERT type 3 Indicator that the structure quality may be low
   1 ALERT type 4 Improvement, methodology, query or suggestion
   2 ALERT type 5 Informative message, check
```

Datablock: 3

```
Bond precision:  C-C = 0.0032 A         Wavelength=0.71073

Cell:           a=10.1719(2)    b=9.5855(2)        c=15.4358(3)
                alpha=90        beta=105.778(1)    gamma=90
Temperature:    296 K

                Calculated                 Reported
Volume          1448.33(5)                 1448.33(5)
Space group     P 21/c                     P 21/c
Hall group      -P 2ybc                    -P 2ybc
Moiety formula  2(C11 H14 Mg N O8), O      ?
Sum formula     C22 H28 Mg2 N2 O17         C22 H30 Mg2 N2 O17
Mr              641.08                     643.10
Dx,g cm-3       1.470                      1.475
Z               2                          2
Mu (mm-1)       0.165                      0.165
F000            668.0                      672.0
F000'           668.59
h,k,lmax        12,11,19                   12,11,19
Nref            2848                       2845
Tmin,Tmax       0.980,0.984                0.702,0.746
Tmin'           0.968

Correction method= # Reported T Limits: Tmin=0.702 Tmax=0.746
AbsCorr = MULTI_SCAN

Data completeness= 0.999          Theta(max)= 26.000

R(reflections)= 0.0520( 2236)     wR2(reflections)= 0.1586( 2845)

S = 1.047                   Npar= 198
```

The following ALERTS were generated. Each ALERT has the format
test-name_ALERT_alert-type_alert-level.
Click on the hyperlinks for more details of the test.

Alert level C

```
PLAT041_ALERT_1_C Calc. and Reported SumFormula    Strings  Differ     Please Check
PLAT043_ALERT_1_C Calculated and Reported Mol. Weight Differ by ..       2.02 Check
```

```
PLAT068_ALERT_1_C Reported F000 Differs from Calcd (or Missing)...     Please Check
PLAT245_ALERT_2_C U(iso) H7C     Smaller than U(eq) O7      by ...      0.022 AngSq
PLAT245_ALERT_2_C U(iso) H7D     Smaller than U(eq) O7      by ...      0.022 AngSq
PLAT250_ALERT_2_C Large U3/U1 Ratio for Average U(i,j) Tensor ....        2.5 Note
PLAT918_ALERT_3_C Reflection(s) with I(obs) much Smaller I(calc) .          1 Check
PLAT975_ALERT_2_C Check Calcd Residual Density  1.09A From      O9       0.73 eA-3
PLAT976_ALERT_2_C Check Calcd Residual Density  1.02A From      O7      -0.49 eA-3
PLAT977_ALERT_2_C Check the Negative Difference Density on     H6D      -0.39 eA-3
PLAT977_ALERT_2_C Check the Negative Difference Density on     H7C      -0.46 eA-3
```

Alert level G

```
FORMU01_ALERT_2_G  There is a discrepancy between the atom counts in the
            _chemical_formula_sum and the formula from the _atom_site* data.
            Atom count from _chemical_formula_sum:C22 H30 Mg2 N2 O17
            Atom count from the _atom_site data:  C22 H28 Mg2 N2 O17
CELLZ01_ALERT_1_G Difference between formula and atom_site contents detected.
CELLZ01_ALERT_1_G WARNING: H atoms missing from atom site list. Is this intentional?
           From the CIF: _cell_formula_units_Z    2
           From the CIF: _chemical_formula_sum  C22 H30 Mg2 N2 O17
           TEST: Compare cell contents of formula and atom_site data

           atom    Z*formula  cif sites diff
           C         44.00     44.00    0.00
           H         60.00     56.00    4.00
           Mg         4.00      4.00    0.00
           N          4.00      4.00    0.00
           O         34.00     34.00    0.00
PLAT004_ALERT_5_G Polymeric Structure Found with Maximum Dimension          3 Info
PLAT007_ALERT_5_G Number of Unrefined Donor-H Atoms ..............          7 Report
PLAT300_ALERT_4_G Atom Site Occupancy of <O9     is Constrained at       0.25 Check
PLAT300_ALERT_4_G Atom Site Occupancy of <O9’    is Constrained at       0.25 Check
PLAT302_ALERT_4_G Anion/Solvent Disorder ............ Percentage =        100 Note
PLAT311_ALERT_2_G Isolated Disordered Oxygen Atom (No H’s ?) .....         O9 Check
PLAT720_ALERT_4_G Number of Unusual/Non-Standard Labels ..........          1 Note
PLAT910_ALERT_3_G Missing # of FCF Reflection(s) Below Theta(Min)           2 Note
PLAT933_ALERT_2_G Number of OMIT records in Embedded RES .........          2 Note
PLAT955_ALERT_1_G Reported (CIF) and Actual (FCF) Lmax Differ by .          1 Units
PLAT978_ALERT_2_G Number C-C Bonds with Positive Residual Density           8 Note
```

---

0 **ALERT level A** = Most likely a serious problem - resolve or explain
0 **ALERT level B** = A potentially serious problem, consider carefully
11 **ALERT level C** = Check. Ensure it is not caused by an omission or oversight
14 **ALERT level G** = General information/check it is not something unexpected

6 ALERT type 1 CIF construction/syntax error, inconsistent or missing data
11 ALERT type 2 Indicator that the structure model may be wrong or deficient
2 ALERT type 3 Indicator that the structure quality may be low
4 ALERT type 4 Improvement, methodology, query or suggestion
2 ALERT type 5 Informative message, check

It is advisable to attempt to resolve as many as possible of the alerts in all categories. Often the minor alerts point to easily fixed oversights, errors and omissions in your CIF or refinement strategy, so attention to these fine details can be worthwhile. In order to resolve some of the more serious problems it may be necessary to carry out additional measurements or structure refinements. However, the purpose of your study may justify the reported deviations and the more serious of these should normally be commented upon in the discussion or experimental section of a paper or in the "special_details" fields of the CIF. checkCIF was carefully designed to identify outliers and unusual parameters, but every test has its limitations and alerts that are not important in a particular case may appear. Conversely, the absence of alerts does not guarantee there are no aspects of the results needing attention. It is up to the individual to critically assess their own results and, if necessary, seek expert advice.

Publication of your CIF in IUCr journals

A basic structural check has been run on your CIF. These basic checks will be run on all CIFs submitted for publication in IUCr journals (*Acta Crystallographica*, *Journal of Applied Crystallography*, *Journal of Synchrotron Radiation*); however, if you intend to submit to *Acta Crystallographica Section C* or *E* or *IUCrData*, you should make sure that full publication checks are run on the final version of your CIF prior to submission.

Publication of your CIF in other journals

Please refer to the *Notes for Authors* of the relevant journal for any special instructions relating to CIF submission.

PLATON version of 11/08/2016; check.def file version of 04/08/2016

Datablock 1 - ellipsoid plot

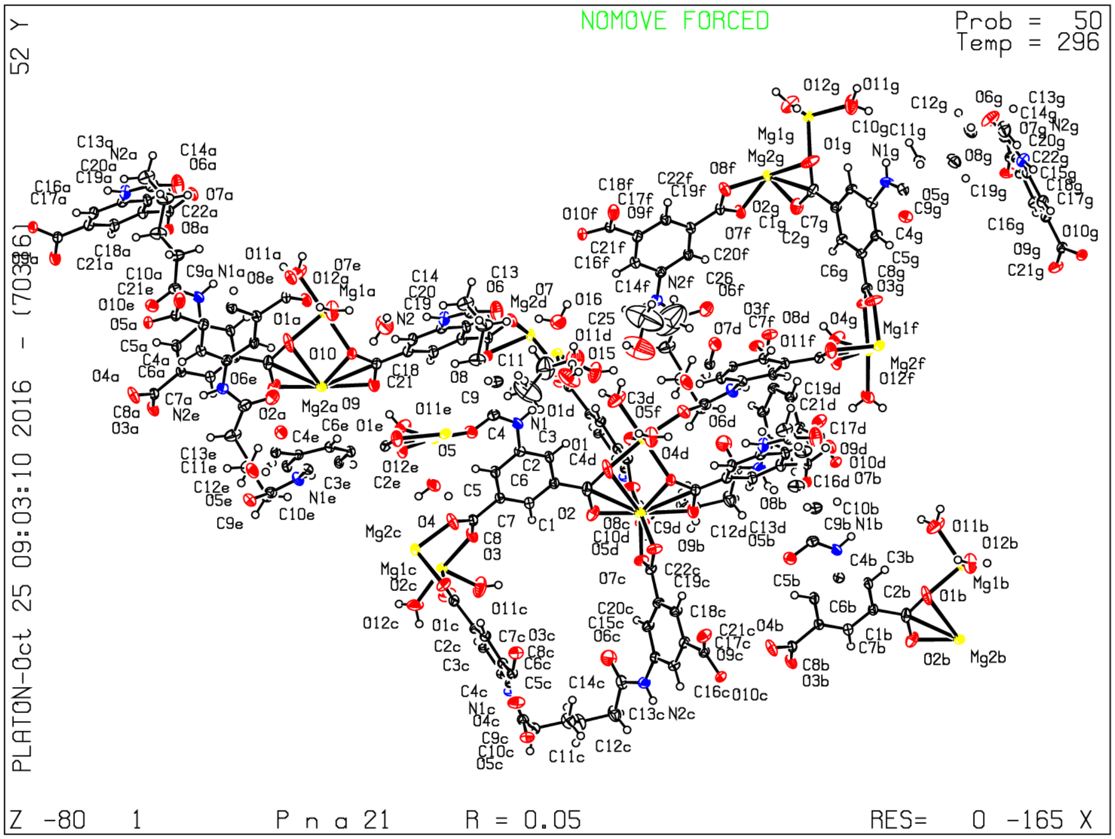

Datablock 2 - ellipsoid plot

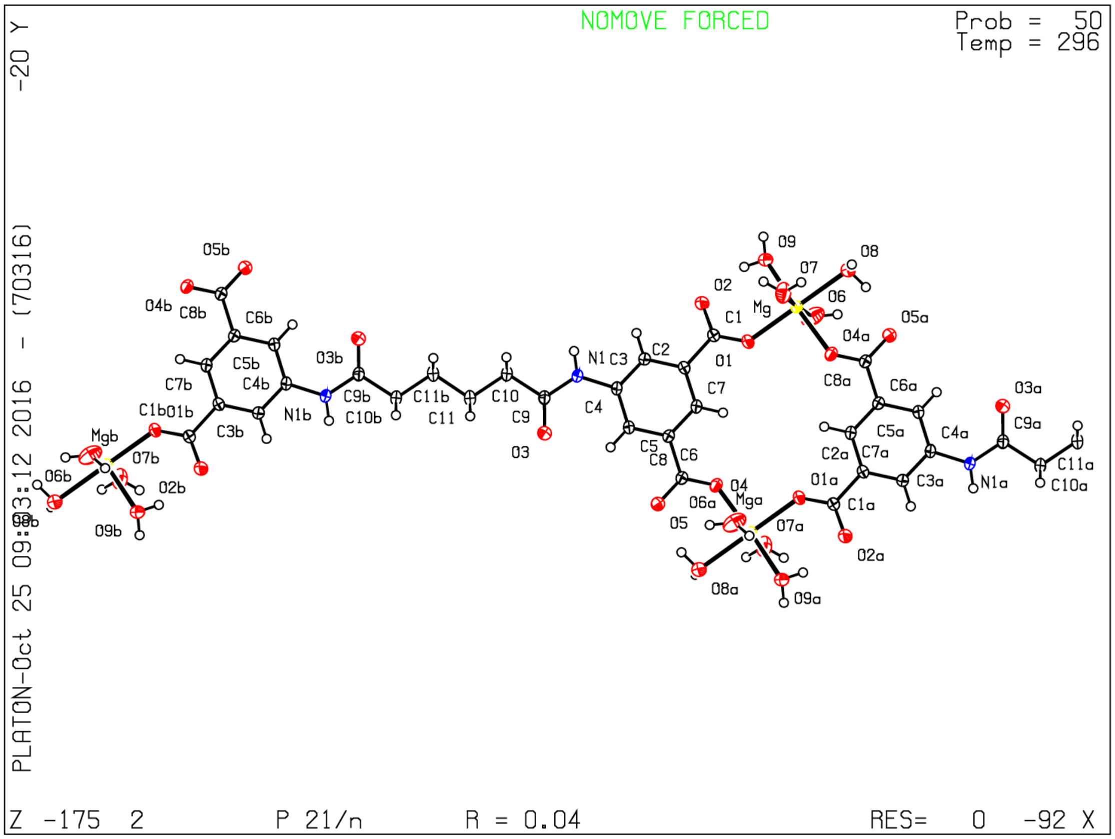

Datablock 3 - ellipsoid plot

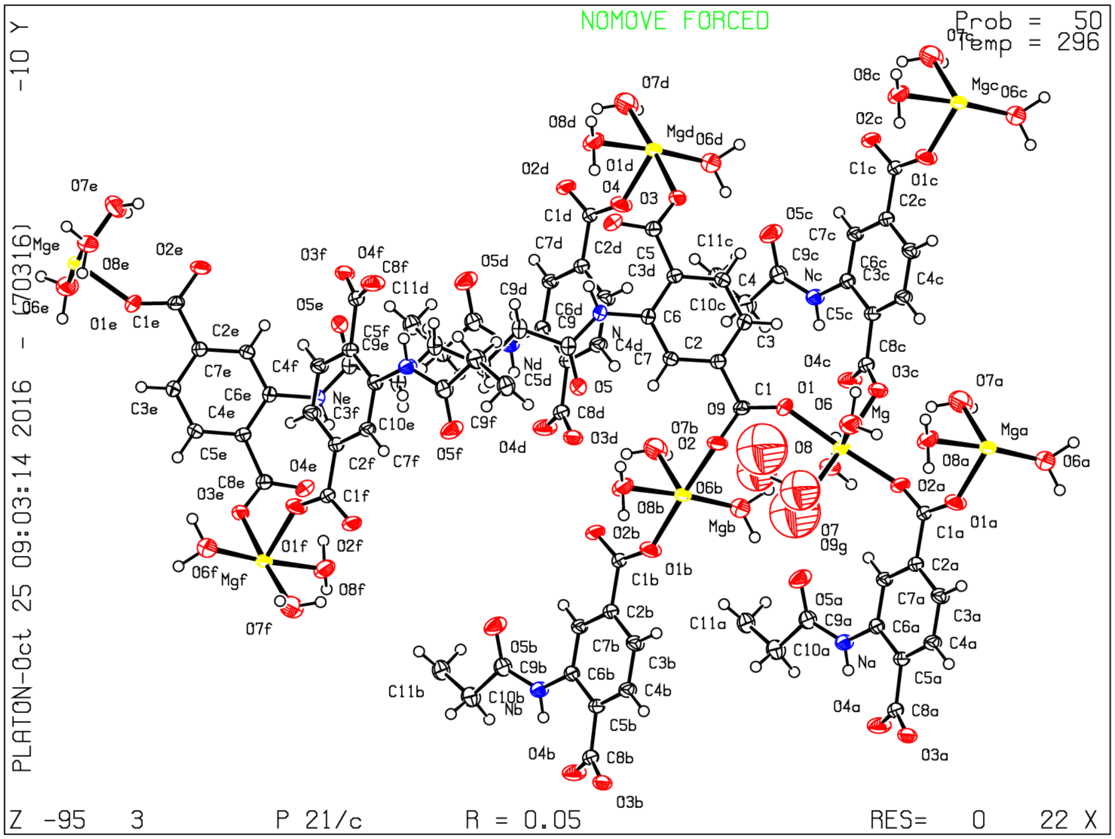

Supplement: Supplementary file 1 [file polymers-10-00371-s001.zip › checkcif.pdf]
