# Supplementary material for: Mg(II) Coordination Polymers Based on Flexible Isomeric Tetracarboxylate Ligands: Syntheses, Structures, Structural Transformation and Luminescent Properties
Source: Polymers (Basel). 2018 Mar 26;10(4):371. doi: 10.3390/polym10040371 (PMC6415218; doi:10.3390/polym10040371)
Supplement: Supplementary file 1 [file polymers-10-00371-s001.zip › supplementary material.docx]

**Supplementary Materials**

**Mg(II) coordinaiton polymers based on flexible isomeric tetracarboxylate igands: synthesis, strutures, thermal, structural transformation and luminescent properties.**

Kedar Bahadur Thapa, Xiang-Kai Yang and Jhy-Der Chen*

*Department of Chemistry, Chung-Yuan Christian University, Chung-Li,*

*Taiwan, R.O.C.*

**Figure S1.** ^1^H NMR (DMSO-d_6_, 400 MHz) of H_4_**L^1^**.


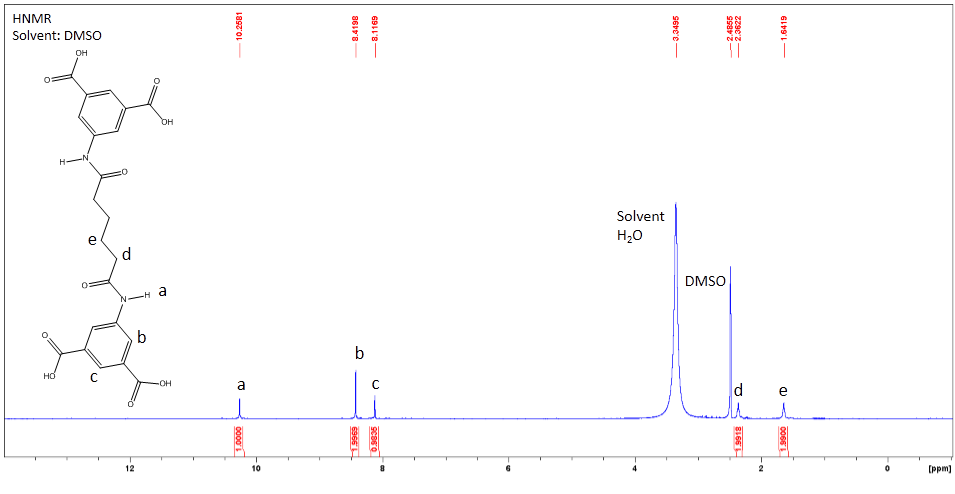


**Figure S2.** ^13^C NMR (DMSO-d_6_, 400 MHz) of H_4_**L^1^**.


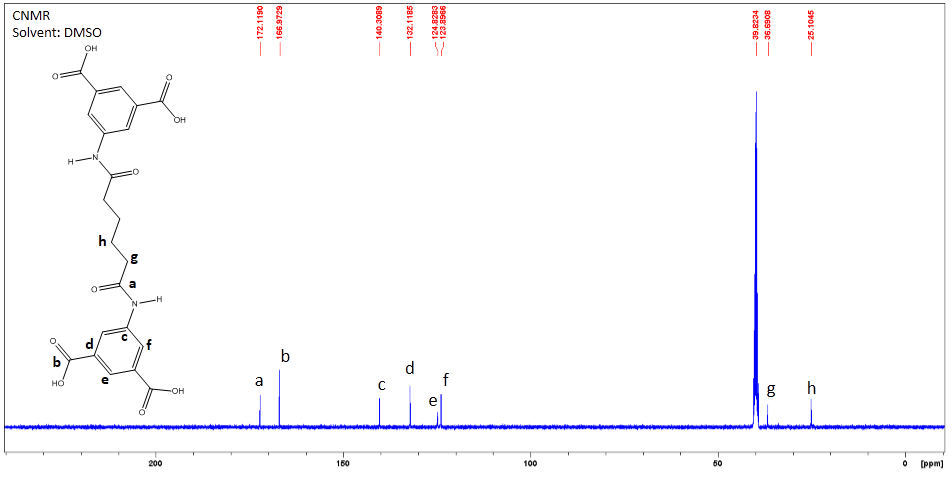


**Figure S3.** TOF, ESI-MS (methanol) of H_4_**L^1^**.

**
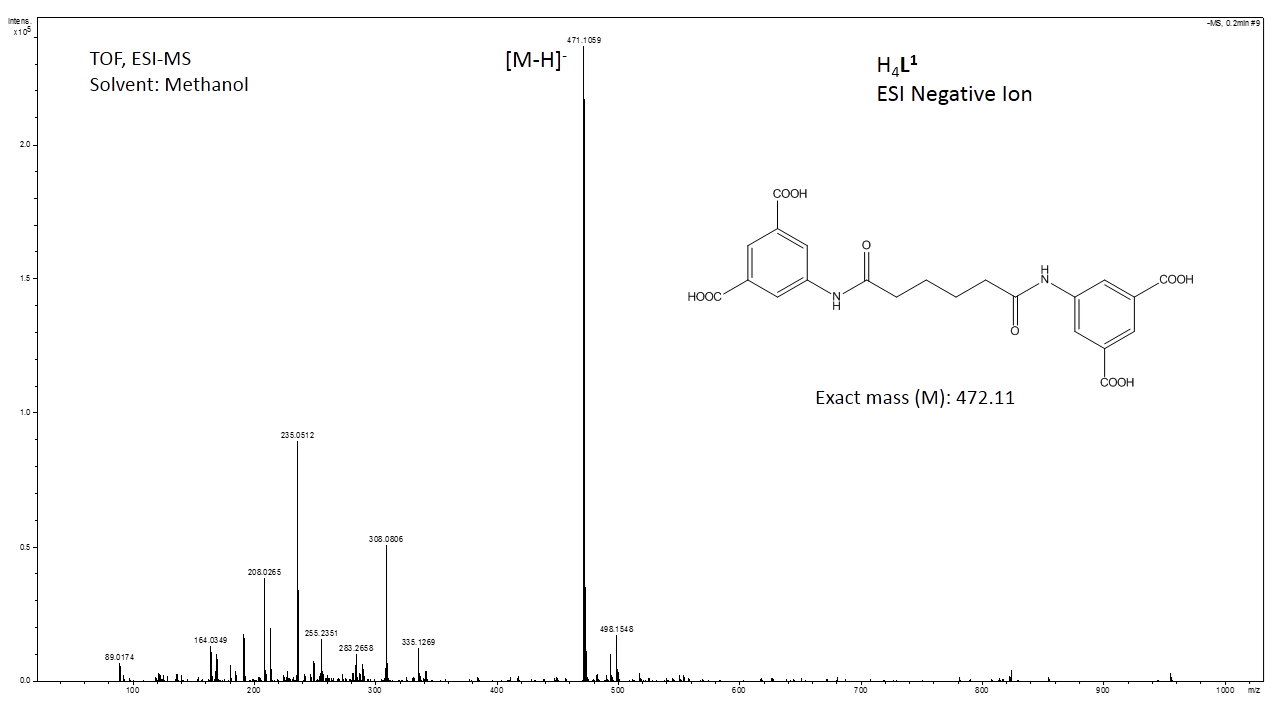
**

**Figure S4.** ^1^H NMR (DMSO-d_6_, 400 MHz) of H_4_**L^2^**.

**
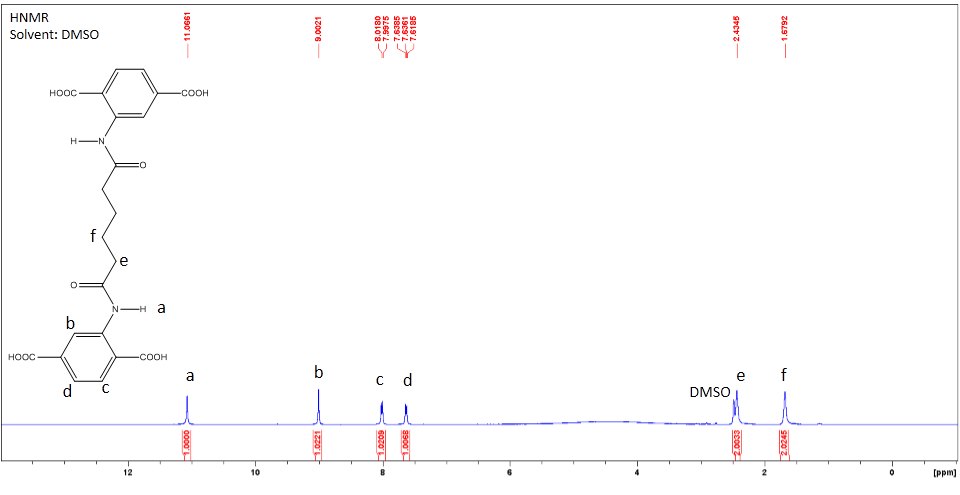
**

**Figure S5.** ^13^C NMR (DMSO-d_6_, 400 MHz) of H_4_**L^2^**.

**
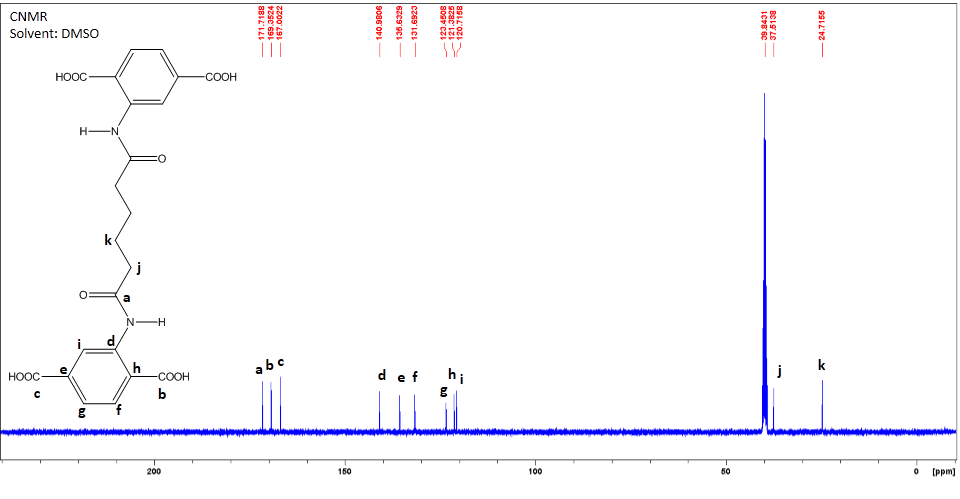
**

**Figure S6.** TOF, ESI-MS (methanol) of H_4_**L^2^**.


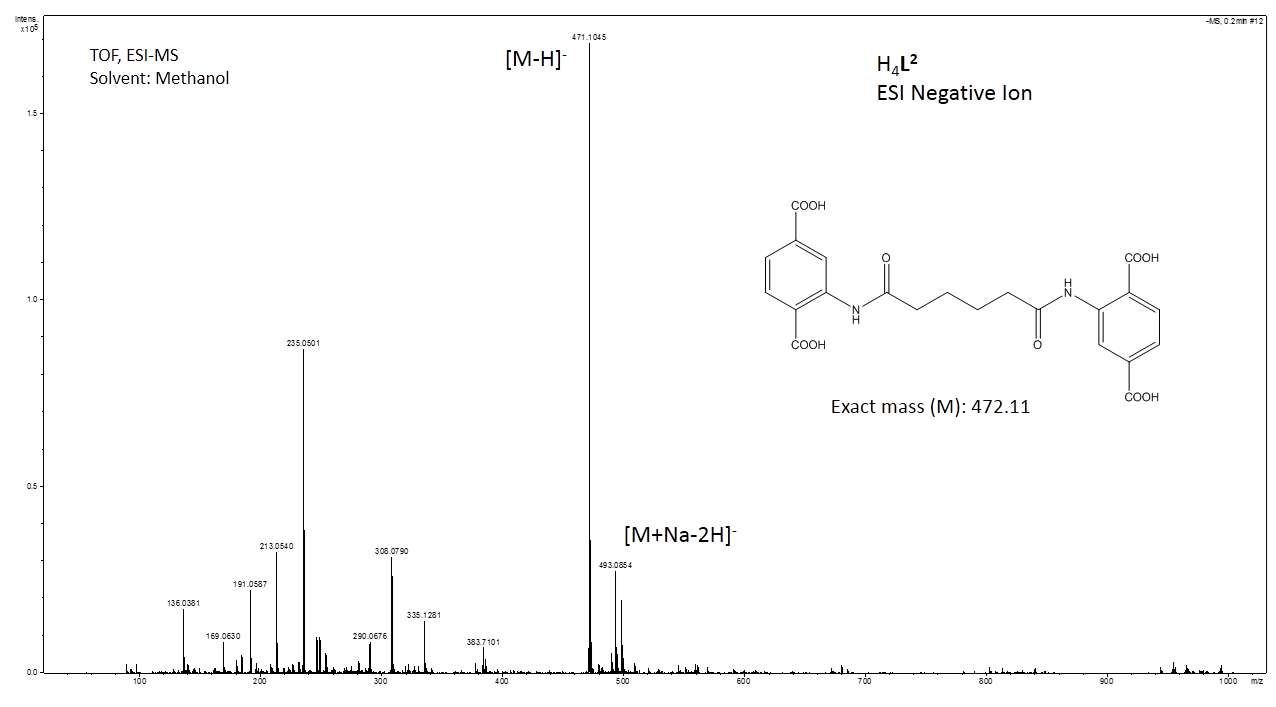


**Figure S7.** Two Mg(II) metal centers acting as a 5-connected single node in **3**.

**
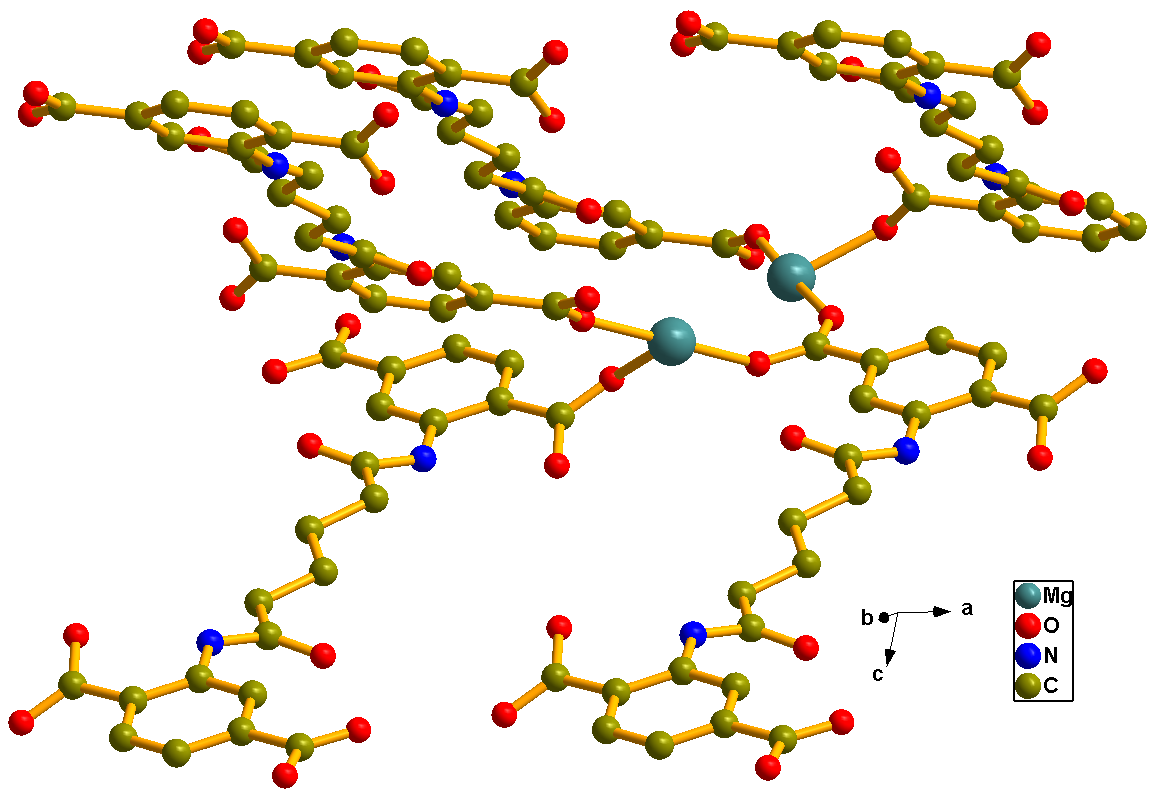
**

**Figure S8.** Simulated and experimental powder X-ray patterns for **1**.

**Figure S9.** Simulated and experimental powder X-ray patterns for **2**.

**Figure S10.** Simulated and experimental powder X-ray patterns for **3**.

**Figure S11.** TGA curves of (a) **1**-**3**.

**Figure S12.** Variable temperature powder XRD patterns of **3** showing irreversible structural transformation.

**Figure S13.** Solid state UV/Visible spectra of **1**-**3**.

**
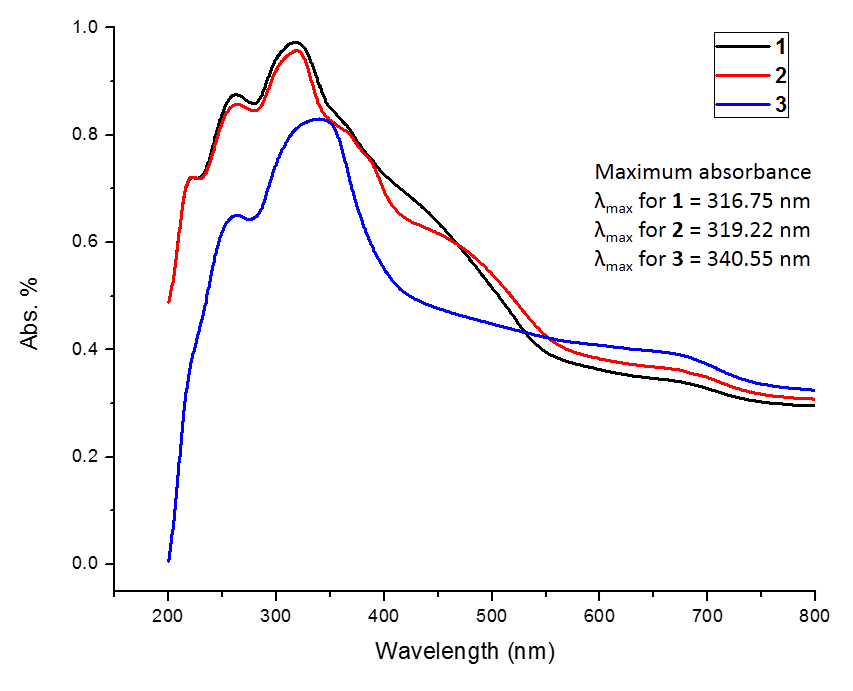
**

**Figure S14.** Solution emission/excitation spectra of H_4_**L^1^** ligand (0.5 mM) in (a) EtOH and (b) DMF.

Ligand Solvent Ex (nm) Em (nm)

H_4_**L^1^** EtOH **262**, 308 353

Ligand Solvent Ex (nm) Em (nm)

H_4_**L^1^** DMF 281, **292**, 327 351

**Figure S15.** Solution emission/excitation spectra of H_4_**L^2^** ligand (0.5 mM) in (a) EtOH and (b) DMF.

Ligand Solvent Ex (nm) Em (nm)

H_4_**L^2^** EtOH 264, **320** 389

Ligand Solvent Ex (nm) Em (nm)

H_4_**L^2^** DMF 278, 299, **352** 388

**Table S1** Selected bond distance (Å) and Angles (^o^) for complexes **1 – 3**

| **1** |  |  |  |
| --- | --- | --- | --- |
| *Distances* |  |  |  |
| Mg(1)-O(1) | 2.020(3) | Mg(2)-O(4B) | 1.996(3) |
| Mg(1)-O(11) | 2.026(4) | Mg(2)-O(2) | 2.070(3) |
| Mg(1)-O(5A) | 2.070(3) | Mg(2)-O(10C) | 2.108(3) |
| Mg(1)-O(12) | 2.073(3) | Mg(2)-O(8D) | 2.112(3) |
| Mg(1)-O(3B) | 2.083(3) | Mg(2)-O(7D) | 2.134(3) |
| Mg(1)-O(10C) | 2.116(3) | Mg(2)-O(9C) | 2.306(3) |
|  |  | Mg(2)-O(1) | 2.725(3) |
| *Angles* |  |  |  |
| O(1)-Mg(1)-O(11) | 95.19(18) | O(4B)-Mg(2)-O(8D) | 163.56(12) |
| O(1)-Mg(1)-O(5A) | 171.93(15) | O(2)-Mg(2)-O(8D) | 97.31(14) |
| O(11)-Mg(1)-O(5A) | 91.11(16) | O(10C)-Mg(2)-O(8D) | 92.48(11) |
| O(1)-Mg(1)-O(12) | 88.91(15) | O(4B)-Mg(2)-O(7D) | 103.13(11) |
| O(11)-Mg(1)-O(12) | 87.86(16) | O(2)-Mg(2)-O(7D) | 95.48(13) |
| O(5A)-Mg(1)-O(12) | 86.28(13) | O(10C)-Mg(2)-O(7D) | 139.38(12) |
| O(1)-Mg(1)-O(3B) | 88.69(14) | O(8D)-Mg(2)-O(7D) | 61.81(10) |
| O(11)-Mg(1)-O(3B) | 87.68(14) | O(4B)-Mg(2)-O(9C) | 87.41(14) |
| O(5A)-Mg(1)-O(3B) | 96.62(12) | O(2)-Mg(2)-O(9C) | 177.43(15) |
| O(12)-Mg(1)-O(3B) | 174.73(14) | O(10C)-Mg(2)-O(9C) | 58.97(10) |
| O(1)-Mg(1)-O(10C) | 84.25(13) | O(8D)-Mg(2)-O(9C) | 85.14(13) |
| O(11)-Mg(1)-O(10C) | 179.04(17) | O(7D)-Mg(2)-O(9C) | 86.34(11) |
| O(5A)-Mg(1)-O(10C) | 89.38(11) | O(4B)-Mg(2)-O(1) | 87.82(12) |
| O(12)-Mg(1)-O(10C) | 91.34(13) | O(2)-Mg(2)-O(1) | 51.96(11) |
| O(3B)-Mg(1)-O(10C) | 93.09(11) | O(10C)-Mg(2)-O(1) | 68.74(9) |
| O(4B)-Mg(2)-O(2) | 90.40(15) | O(8D)-Mg(2)-O(1) | 108.37(12) |
| O(4B)-Mg(2)-O(10C) | 96.21(12) | O(7D)-Mg(2)-O(1) | 146.15(12) |
| O(2)-Mg(2)-O(10C) | 119.99(12) | O(9C)-Mg(2)-O(1) | 126.54(10) |
|  |  |  |  |
| **2** |  |  |  |
| *Distances* |  |  |  |
| Mg-O(1) | 2.046(1) | Mg-O(7) | 2.081(1) |
| Mg-O(4A) | 2.051(1) | Mg-O(6) | 2.089(1) |
| Mg-O(9) | 2.059(1) | Mg-O(8) | 2.186(1) |
|  |  | Mg(A) -O(4) | 2.051(1) |
| *Angles* |  |  |  |
| O(1)-Mg-O(4A) | 91.78(4) | O(9)-Mg-O(6) | 85.82(5) |
| O(1)-Mg-O(9) | 90.57(5) | O(7)-Mg-O(6) | 174.98(6) |
| O(4A)-Mg-O(9) | 171.75(6) | O(1)-Mg-O(8) | 179.12(6) |
| O(1)-Mg-O(7) | 92.04(6) | O(4A)-Mg-O(8) | 88.04(4) |
| O(4A)-Mg-O(7) | 95.49(5) | O(9)-Mg-O(8) | 89.73(5) |
| O(9)-Mg-O(7) | 92.33(6) | O(7)-Mg-O(8) | 87.12(5) |
| O(1)-Mg-O(6) | 92.65(6) | O(6)-Mg-O(8) | 88.20(6) |
| O(4A)-Mg-O(6) | 86.17(5) |  |  |
|  |  |  |  |
| **3** |  |  |  |
| *Distances* |  |  |  |
| Mg-O(2A) | 2.0141(19) | Mg-O(3B) | 2.0934(19) |
| Mg-O(1) | 2.0316(19) | Mg-O(8) | 2.130(2) |
| Mg-O(6) | 2.059(2) | O(2)-Mg(C) | 2.0141(19) |
| Mg-O(7) | 2.085(2) | O(3)-Mg(D) | 2.0934(19) |
|  |  |  |  |
| *Angles* |  |  |  |
| O(2A)-Mg-O(1) | 176.98(8) | O(6)-Mg-O(3B) | 89.61(8) |
| O(2A)-Mg-O(6) | 89.86(9) | O(7)-Mg-O(3B) | 176.70(9) |
| O(1)-Mg-O(6) | 89.00(9) | O(2A)-Mg-O(8) | 93.83(9) |
| O(2A)-Mg-O(7) | 90.97(9) | O(1)-Mg-O(8) | 87.19(9) |
| O(1)-Mg-O(7) | 91.88(9) | O(6)-Mg-O(8) | 175.55(8) |
| O(6)-Mg-O(7) | 92.34(11) | O(7)-Mg-O(8) | 90.09(10) |
| O(2A)-Mg-O(3B) | 91.70(8) | O(3B)-Mg-O(8) | 87.80(8) |
| O(1)-Mg-O(3B) | 85.49(8) |  |  |
|  |  |  |  |

Symmetry transformations used to generate equivalent atoms:

(A) -x+3/2,y+1/2,z+1/2, (B) -x+1,-y+1,z+1/2, (C) x,y+1,z, (D) -x+1,-y+1,z-1/2 for **1**.

(A) -x,-y,-z for **2**. (A) -x,y-1/2,-z+1/2, (B) -x+1,y-1/2,-z+1/2, (C) -x, y+1/2,-z+1/2,

(D) -x+1,y+1/2,-z+1/2 for **3**.
